# Modified-Live Feline Calicivirus Vaccination Elicits Cellular Immunity against a Current Feline Calicivirus Field Strain in an Experimental Feline Challenge Study

**DOI:** 10.3390/v13091736

**Published:** 2021-08-31

**Authors:** Andrea M. Spiri, Marilisa Novacco, Marina L. Meli, Martina Stirn, Barbara Riond, Jonathan E. Fogle, Felicitas S. Boretti, Imogen Herbert, Margaret J. Hosie, Regina Hofmann-Lehmann

**Affiliations:** 1 Clinical Laboratory, Department of Clinical Diagnostics and Services, Center for Clinical Studies, Vetsuisse Faculty, University of Zurich, CH-8057 Zurich, Switzerland; mnovacco@vetclinics.uzh.ch (M.N.); mmeli@vetclinics.uzh.ch (M.L.M.); mstirn@vetclinics.uzh.ch (M.S.); briond@vetclinics.uzh.ch (B.R.); rhofmann@vetclinics.uzh.ch (R.H.-L.); 2 Comparative Immunology Research Laboratory, College of Veterinary Medicine, North Carolina State University, Raleigh, NC 27695, USA; jonathan.fogle@boehringer-ingelheim.com; 3 Clinic for Small Animal Internal Medicine, Vetsuisse Faculty, University of Zurich, CH-8057 Zurich, Switzerland; fboretti@vetclinics.uzh.ch; 4 Medical Research Council-University of Glasgow, Centre for Virus Research, Glasgow G61 1QH, UK; imogen.herbert@glasgow.ac.uk (I.H.); margaret.hosie@glasgow.ac.uk (M.J.H.)

**Keywords:** crossneutralisation, crossimmunity, lymphocyte subsets, neutralising antibodies, cytokines, IFN-γ, perforin, granzyme B, antiviral factor MX1, ELISpot

## Abstract

Feline calicivirus (FCV) is a common cat virus associated with oral ulcerations and virulent-systemic disease. Efficacious FCV vaccines protect against severe disease but not against infection. The high genetic diversity of FCV poses a challenge in vaccine design. Protection against FCV has been related to humoral and cellular immunity; the latter has not been studied in detail. This study investigates the cellular and humoral immune response of specified pathogen-free (SPF) cats after modified-live FCV F9 vaccinations and two heterologous FCV challenges by the analysis of lymphocyte subsets, cytokine mRNA transcription levels, interferon (IFN)-γ release assays in peripheral blood mononuclear cells (PBMCs), anti-FCV antibodies, and neutralisation activity. Vaccinated cats developed a Th1 cytokine response after vaccination. Vaccination resulted in antibodies with neutralising activity against the vaccine but not the challenge viruses. Remarkably, IFN-γ-releasing PBMCs were detected in vaccinated cats upon stimulation with the vaccine strain and the first heterologous FCV challenge strain. After the first experimental infection, the mRNA transcription levels of perforin, granzyme B, INF-γ, and antiviral factor MX1 and the number of IFN-γ-releasing PBMCs when stimulated with the first challenge virus were higher in vaccinated cats compared to control cats. The first FCV challenge induced crossneutralising antibodies in all cats against the second challenge virus. Before the second challenge, vaccinated cats had a higher number of IFN-γ-releasing PBMCs when stimulated with the second challenge virus than control cats. After the second FCV challenge, there were less significant differences detected between the groups regarding lymphocyte subsets and cytokine mRNA transcription levels. In conclusion, modified-live FCV vaccination induced cellular but not humoral crossimmunity in SPF cats; innate immune mechanisms, secretory and membranolytic pathways, and IFN-γ-releasing PBMCs seem to be important in the host immune defence against FCV.

## 1. Introduction

FCV is one of the most common viral pathogens in cats worldwide [1]. FCV prevalence can range from low to high (10–90%) [2,3,4,5,6,7], depending on the population sampled, but multicat situations, such as in shelters or breeding catteries, are especially of concern. Typical clinical signs consist of oral ulcerations, fever, and reduced general condition, and some FCV strains can also cause pneumonia or limping [1,7,8]. FCV can also present as a virulent-systemic disease with extensive mucosal and skin ulcerations, subcutaneous oedema, and inner organ involvement, which can lead to high morbidity and lethality [9,10,11]. FCV is a single-stranded RNA virus belonging to the family Caliciviridae, genus Vesivirus, and due to the lack of proofreading of the viral polymerase, the viral evolution rate is very high [1,12], which presents a challenge for efficient and broadly acting FCV vaccines [13]. Currently, two types of FCV vaccines are mainly used in Europe: (1) A modified-live single-strain vaccine containing FCV F9 or (2) an inactivated double-strain vaccine containing FCV 431 and FCV G1. Both types of FCV vaccines do not induce sterilising immunity but rather reduce the severity of clinical signs if cats are affected by a classical FCV disease [13]. For cats suffering from virulent-systemic (VS-)FCV, vaccination was not protective against severe disease in several cases [11,14,15]. Some studies show a protective effect of FCV vaccination in experimentally induced VS-FCV disease [10], and another study reported that many VS-FCV-affected cats were insufficiently vaccinated [9]. It was demonstrated that the level of neutralising antibodies correlates with protection against homologous challenge [16]. However, cats without neutralising antibodies were also found to be protected against disease [17,18,19,20]. The neutralisation potential of FCV F9 after decades of use in the feline population has been controversial. Some studies suggest that the prolonged use of the FCV vaccine strain F9 might have driven the evolution of circulating FCV strains towards vaccine-resistant variants [21,22], whereas other studies do not confirm a divergence of field viruses from the vaccine strain F9 [23,24]. The protection of cats lacking neutralising antibodies implies an important role for cellular immunity in FCV infection. A study from Tham and Studdert (1987) [25] detected cellular immune response mechanisms in cats after an inactivated FCV vaccination and homotypic FCV challenge, and FCV-specific CD4^+^ T-cells were detected in the spleens of cats vaccinated with a modified-live FCV vaccine [26]. Thus far, a comprehensive study on the humoral and cellular immune response of cats after FCV F9 vaccination and after two subsequent experimental FCV infections with recently collected field strains is lacking.

The aim of the present study was to provide a comprehensive characterisation of a cat’s immune response after vaccination with a modified-live (MLV) FCV vaccine containing FCV F9 and after two subsequent heterologous FCV challenge infections with recently collected FCV field strains. The immune response was assessed in vaccinated cats and compared to unvaccinated controls by means of lymphocyte subset characterisation, the analysis of cytokine mRNA transcription levels and IFN-γ release assay, specific antibody response, and virus neutralisation activity.

## 2. Materials and Methods

### 2.1. Study Setup

The study setup is described in detail in Spiri et al. (2019) [27], and Spiri et al. (2021) [28]. Briefly, ten SPF cats were divided in two groups: five cats (cat ID: JJG4, JJG6, JJH3, JJI1, and JJI2) were vaccinated subcutaneously twice 21 days apart at 15 and 18 weeks of age using a MLV trivalent vaccine containing FCV F9, and five cats (cat ID: JJF1, JJG3, JJH2, JJI3, and JJI4) served as a control group and received a placebo injection (FCV Vaccination I). All cats were infected oronasally with the heterologous FCV field strain FCV 273 (FCV Challenge I) 7 months after the second injection of the first vaccination or placebo vaccination. Subsequently, 11 months after FCV Challenge I, all cats were revaccinated once with the respective vaccine or placebo (FCV Vaccination II), and one month after revaccination (FCV Vaccination II), all cats were infected oronasally with the heterologous FCV field strain FCV 27 (FCV Challenge II).

### 2.2. Blood Collection, Processing, and Analyses

Blood was taken from the vena cephalica without anaesthesia. Ethylenediaminetetraacetic acid (EDTA)-anticoagulated blood was used for flow cytometry and cytokine analyses. Whole blood collected in plain tubes was centrifuged at 1862× *g* for five minutes, and serum aliquots were immediately stored at −80 °C until immunofluorescence assays (IFA) or neutralisation assays were performed. Heparinised blood was used for isolation of peripheral blood mononuclear cells (PBMCs). Blood samples were centrifuged at 176× *g*; plasma was removed and replaced by Hank’s balanced salt solution (HBSS, Gibco, Zug, Switzerland) mixed thoroughly; and PBMCs were isolated by density gradient centrifugation at 703× *g* using Histopaque^®^ 1077 (Sigma-Aldrich, Buchs, Switzerland). Cells were washed in HBSS, gradually frozen (−1 °C/min) in recovery cell culture freezing medium (Gibco) containing DMSO and stored in liquid nitrogen until used for enzyme-linked immunospot (ELISpot) assay. The timepoints of blood collections are shown in Table 1.

### 2.3. FCV Seroreactive Antibodies

IFA slides were coated with FCV F9, and the serum samples were tested as published and described in detail elsewhere [29,30]. Sera of FCV antibody-positive field cats and an FCV-negative SPF cat were used as positive and negative controls. Antibody titres reflect the reciprocal of the last serum dilution showing positive fluorescence in the IFA [29,30].

### 2.4. FCV-Neutralising Antibodies

Virus strains FCV 273 (virus for FCV Challenge I), FCV 27 (virus for FCV Challenge II), a Swiss VS-FCV field isolate (FCV ZH2016), and FCV F9 (virus for FCV Vaccination I and II) were expanded on feline embryo A (FEA) cells and serum-free 1 × Dulbecco’s modified Eagle’s medium (DMEM; Gibco, Paisley, UK) containing 1% L-Glutamine 200 mM (Gibco, UK) and 1% sodium pyruvate 100 mM (Gibco, UK). After complete cytopathic effect (CPE), supernatants and cells were harvested and freeze-thawed three times. Dead cells and debris were pelleted, and the supernatants were further used to determine 50% tissue culture infective dose (TCID_50_)/mL for each virus. From each cat serum and each time point, six serial dilutions were prepared starting at 1:5 and continuing with 3-fold serial dilutions (1:5, 1:15, 1:45, 1:135, 1:405, and 1:1215). All dilutions were made with 1 × DMEM (Gibco, UK) supplemented with 10% foetal bovine serum (FBS, Gibco, UK), 1% L-Glutamine 200 mM (Gibco, UK), 1% sodium pyruvate 100 mM (Gibco, UK), 1% Gentamicin 10 mg/mL (Gibco, UK) and 2.4% penicillin 10,000 U/mL (Gibco, UK), and Streptomycin 10,000 µg/mL (Gibco, UK). Fifty microliters of each serum dilution was transferred in quadruplicate to a 96-well plate. FCV strains were diluted to 100 TCID_50_/mL with DMEM complete, and 50 µL of 100 TCID_50_/mL of each virus and 50 µL of each serum dilution were incubated for 2 h at 37 °C where the antibodies could neutralise the virus. After 2 h, 3 × 10^4^ FEA cells in 150 µL of DMEM complete were added to each well. After 48 h of incubation at 37 °C, each well was assessed microscopically for CPE. The titre was the reciprocal of the serum dilution where 50% or more of the wells showed a CPE. Positive (virus and cells only) and negative (cells only) controls were run in parallel on the same plate. A titre >7 was considered to be protective [16].

### 2.5. Cytokines

For the measurement of cytokine mRNA transcription levels, 100 µL EDTA anticoagulated blood was transferred to 700 µL of lysis buffer from the MagNA Pure LC RNA isolation kit–high performance (Roche Diagnostics AG, Rotkreuz, Switzerland), transiently stored on ice immediately after blood collection, and stored at −80 °C within 3 h of collection until further use. Total RNA was extracted using the MagNA Pure LC RNA isolation kit–high performance (Roche Diagnostics AG) according to the manufacturer’s instructions. RNA was transcribed to cDNA using the high-capacity cDNA reverse-transcription kit (Applied Biosystems, Zug, Switzerland). The mRNA expression for the following cytokines was measured by real-time quantitative polymerase chain reaction (qPCR) as described [31,32]: interleukin (IL)-4, IL-6, IL-10, IL12p40, tumour necrosis factor (TNF)-α, interferon (IFN)-γ, IFN-α, IFN-β, IFN-ω, antiviral factor MX1, perforin, and granzyme B. Standard curves and negative controls were run in parallel with each assay. IL-12p40 and IFN-γ were measured to determine an immune response indicating activation of the T-helper (Th)1 lineage, and IL-4, IL-6, and IL-10 were measured to determine a Th2-directed response. TNF-α together with IL-6 were used to measure proinflammatory responses, and Il-10 is an anti-inflammatory cytokine. Type I IFNs were characterised by IFN-α, IFN-β and IFN-ω, and antiviral factor MX1 was determined. The secretory and membranolytic pathway was represented by perforin and granzyme B. Zeta polypeptide (YWHAZ) and V-abl Abelson murine leukaemia viral oncogene homolog (ABL) were used as reference genes for normalisation in feline peripheral blood [33]. Calculation of mRNA transcription levels was performed using GeNorm as described [34].

### 2.6. Lymphocyte Subsets

Four different staining protocols for lymphocyte subset analyses were used: (1) fluorescein isothiocyanate (FITC)-conjugated mouse antifeline CD5 antibody (f43, Southern Biotech, Allschwil, Switzerland), MHCII unconjugated IgG2b mouse antifeline (H34A, VMRD, Pullmann, WA, USA), and R-phycoerythrin (RPE)-conjugated goat antimouse IgG2b (Southern, Biotech) as a secondary antibody; (2) an R-phycoerythrin (PE)-conjugated mouse antifeline CD4 antibody (3-4F4, Southern Biotech) and an APC-conjugated mouse antifeline CD25 (9F23) antibody (courtesy of J.E. Fogle, North Carolina State University, Raleigh, NC, USA); and (3) an FITC-conjugated mouse antifeline CD8 antibody (fCD8, Southern Biotech) and (4) a PE-conjugated rat antimouse CD45R/B220 antibody (RA3-6B2, Bio-Rad, Cressier, Switzerland). CD4^+^- and CD8^+^-cells represent helper and cytotoxic T-cells, respectively [35]. CD4^+^CD25^+^ and CD5^+^/MHCII^+^ cells represent activated T-cells [36], and CD45R/B220 antibody was used to identify B-cells [37,38].

The antibodies were titrated prior to the start of the experiment. CD4 was used at a 1:250 dilution, CD8 at a 1:500 dilution, MHCII and secondary IgG2b at a 1:1000 dilution, CD25 at a 1:50 dilution, and CD45R/B220 at a 1:25 dilution. Negative controls consisted of unstained samples. Dead cells were excluded from the analyses using fixable viability dye eFluor 780 (eBioscience, San Diego, CA, USA). Blood samples, consisting of 50 µL, were stained according to published protocols [39,40]. The stainings for CD25 and MHC II were included in the flow cytometry panel from FCV Challenge I on.

Flow cytometry was performed using a BD FACSCanto II™ Flow Cytometer (Becton, Dickinson and Company Biosciences, Allschwil, Switzerland) at the flow cytometry facility in the University of Zurich, Switzerland. The leukocyte population was brought into focus based on forward and side scatter, and 10,000 events were acquired. The absolute number of each lymphocyte subset was calculated by multiplying the absolute lymphocyte number assessed in haematology [28] with the lymphocyte subset percentage as previously published [41]. The gating strategy consisted of all leukocytes, all lymphocytes, single cells, live cells, and FITC/PE or PE/APC or SSC-A/FITC or SSC-A/PE. Gating was carried out using FlowJo™ (FlowJo™ Software for Windows Version 10.7.1, Becton, Dickinson and Company Biosciences, Allschwil, Switzerland).

### 2.7. Feline IFN-γ ELISpot

PBMCs stored in liquid nitrogen were rapidly thawed in a 37 °C water bath, washed and pelleted twice, and resuspended in sterile Roswell Park Memorial Institute (RPMI) 1640 medium (Gibco) completed with 10% heat-inactivated foetal calf serum (Gibco), 2 mM L-Glutamin (Gibco) and 1 × antibiotic/antimycotic (Sigma-Aldrich). Cell counting was performed with a Sysmex XN-1000V (Sysmex, Horgen, Switzerland) haematology analyser using the body-fluid mode. Gates dividing polymorphnuclear and mononuclear cells were inspected visually and adapted if necessary. The mononuclear cell count was used to calculate the number of PBMCs per well. For IFN-γ detection, the feline IFN-γ ELISpot Kit (R&D Systems, Zug, Switzerland) was used. PBMCs at a concentration of 1–5 × 10^5^ cells/well were incubated with heat-attenuated FCV cell culture supernatants (FCV 273, FCV 27, FCV ZH2016 or FCV F9), and 5 × 10^4^ PBMCs/well were incubated with 1 μg/well Concanavalin A from *Canavalia ensiformis* (conA; Sigma-Aldrich) as a stimulation control or 100 µL RPMI complete/well as a negative control for 48 h at 37 °C in a humidified CO_2_ incubator. Recombinant feline IFN-γ was used as positive control. Cells were left undisturbed during the 48 h incubation period. FCV cell culture supernatants were heat-attenuated for 30 min at 56 °C prior to the incubation with cells. Before heat attenuation, the cell culture supernatants were at the same concentrations as used for the experimental infection as described previously, and FCV F9 supernatant was used at a concentration of 3.16 × 10^8^ TCID_50_/mL. Spot development was performed according to the manufacturer’s instructions. After completion of the assay, the plates were carefully air-dried for at least 24 h. Plate reading and spot counting were performed with an AID classic ELISpot reader V7.0 (Autoimmun Diagnostika GmbH, Strassberg, Germany). Spot counts were mathematically corrected to 5 × 10^5^ cells.

### 2.8. Statistics

All data were compiled in Microsoft^®^ Excel^®^ 2016 for Microsoft 365, version 2103 and analysed using GraphPad Prism 9 for Windows (San Diego, CA, USA). The Fisher’s exact test was applied to test for differences in proportions. The Mann–Whitney U test was used to test for differences between the vaccine and the control group. Friedman’s test and Dunn’s post test tested the changes over time within a group.

## 3. Results

### 3.1. FCV Vaccination I

**Figure 1:**
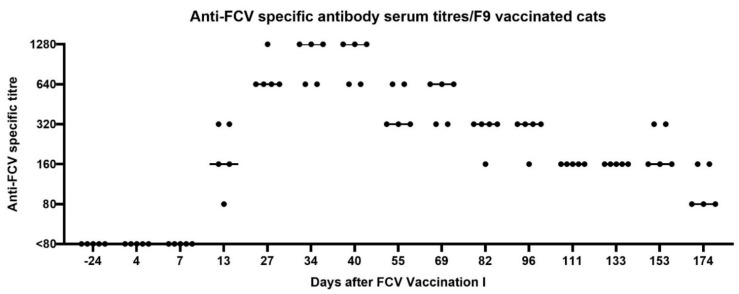
Dot plot of anti-FCV specific antibody serum titres of the five vaccinated cats measured by immunofluorescence assay (IFA) before and after FCV Vaccination I with FCV F9. Each dot represents the titre of a cat of the vaccine group. The horizontal bar represents the median. The second injection of FCV Vaccination I was performed at day 21 after the first injection. Antibody titres reflect the reciprocal of the last serum dilution showing positive fluorescence in the IFA. Days are indicated as days after the first injection of FCV Vaccination I.

After FCV Vaccination I, seroconversion, as determined by a positive IFA titre in the FCV F9-vaccinated cats, occurred between days 7 and 13 after the first injection of FCV Vaccination I, with titres of 80–320 at day 13 (Figure 1). One week after the second injection of FCV Vaccination I, which took place at day 21, titres were increasing up to 640–1280 (Figure 1, day 27). The highest titres (max. 1280) were reached between days 27–40 after FCV Vaccination I. The FCV antibody titres decreased gradually from day 55 to day 174 after FCV Vaccination I, and at day 174, all vaccinated cats had FCV titres of 80 or 160 (Figure 1). All placebo-injected cats tested negative (titre <80) in the IFA assay during the vaccination phase.

**Figure 2:**
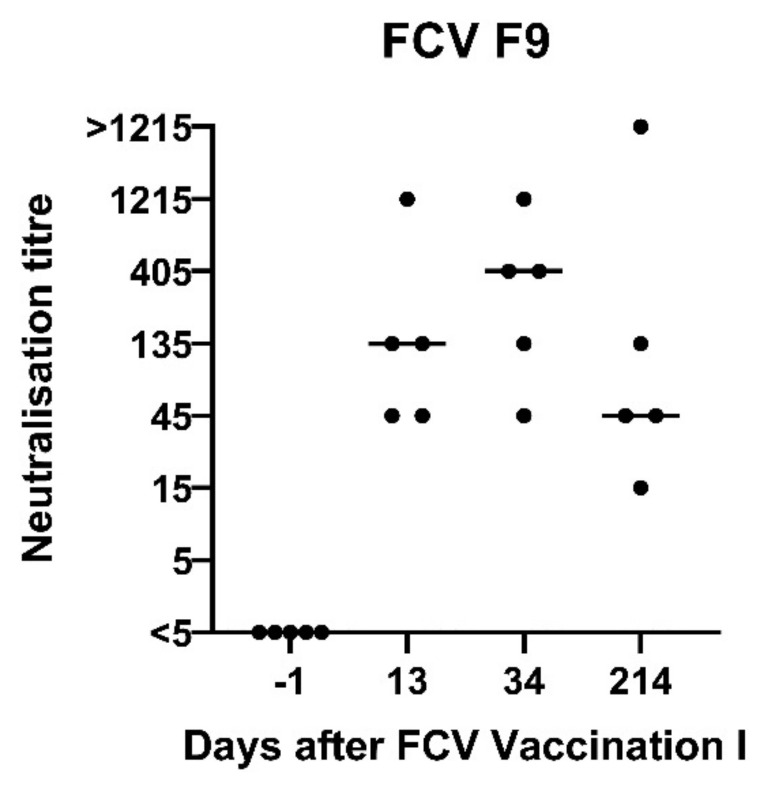
Dot plot of FCV F9 neutralisation titres in vaccinated cats after FCV Vaccination I with FCV F9. The titre is the reciprocal of the serum dilution where 50% or more of the wells show a CPE. Each dot represents the titre of a cat of the vaccine group. The horizontal bar represents the median. Days are indicated as days after the first injection of FCV Vaccination I. The second injection of FCV Vaccination I was performed at day 21 after the first injection.

Neutralising antibodies against FCV F9 were detected at day 13 after the first injection of FCV Vaccination I in FCV F9-vaccinated cats (Figure 2). At day 34 after the first injection (13 days after the second injection), the neutralising antibody titres against FCV F9 increased in two cats and stayed at the same level in three cats. All five control cats receiving a placebo injection stayed seronegative (titre <5) prior to FCV Challenge I. No in vitro crossneutralisation was detected when sera from FCV F9-vaccinated cats were tested against FCV 273, FCV 27 and a Swiss VS-FCV (FCV ZH2016) field strain (titre <5).

**Figure 3:**
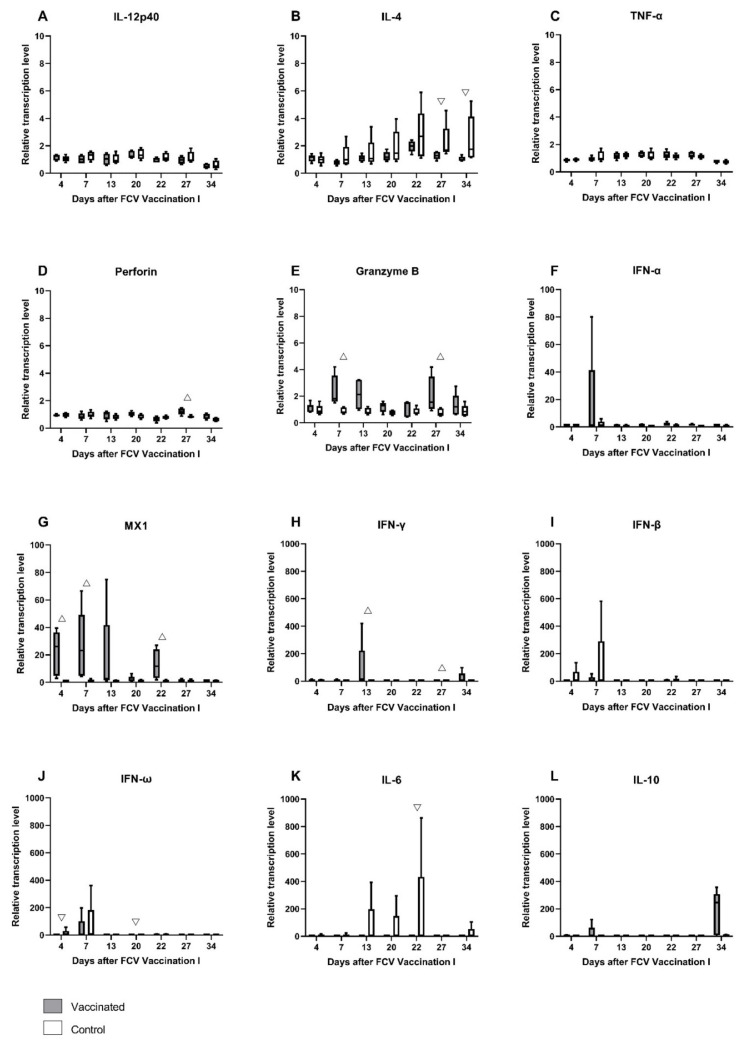
Box and whisker plots of cytokine expression profile of IL-12p40 (**A**), IL-4 (**B**), TNF-α (**C**), perforin (**D**), granzyme B (**E**), IFN-α (**F**), MX1 (**G**), IFN-γ (**H**), IFN-β (**I**), IFN-ω (**J**), IL-6 (**K**) and IL-10 (**L**) after FCV Vaccination I. The line inside the box shows the median, and the lower and upper border of the box indicates the 25th and 75th percentile, respectively. Whiskers represent minimum and maximum values. Significant differences between the vaccinated and the control group are indicated with an open triangle; ▽ denotes significantly lower in the vaccinated group P_MWU_ ≤ 0.05; △ denotes significantly higher in the vaccinated group P_MWU_ ≤ 0.05. The second injection of FCV Vaccination I was performed at day 21. The y-axes are scaled either at 0–10, 0–100, or 0–1000 relative mRNA transcription level, and the graphs are ordered respectively.

Relative mRNA transcription levels of cytokines and proteins of the innate and the adaptive immune system that act pro- or anti-inflammatory or promote either the Th1- or Th2-differentiation of T-cells were investigated in peripheral blood (Figure 3A–L). After the first injection of FCV Vaccination I, the upregulation of mRNA transcription levels of granzyme B, IFN-α, MX-1, IFN-γ, IFN-ω, and IL-10 was detected in FCV-vaccinated cats (Figure 3E–H,J,L). In the control cats, an upregulation of IL-4, IFN-β, IFN-ω, and IL-6 was observed after the first placebo injection (Figure 3B,I–K). After the second injection of FCV Vaccination I, mRNA transcription levels of INF-γ, IL-10, MX1, perforin, and granzyme B were upregulated in the FCV-vaccinated cats, and IL-4 and IL-6 were upregulated in the control cats. The most prominent change in median mRNA transcription levels compared to prevaccination levels was observed in FCV-vaccinated but not in the control cats for IL-10 (up to 245-fold) at day 34 after the first injection of FCV Vaccination I (Figure 3L). For antiviral factor MX1, there was up to a 25-fold increase in median mRNA transcription levels observed in FCV-vaccinated cats at day 4 compared to prevaccination levels. For IFN-γ, the highest median upregulation (14-fold) was observed at day 13 after the first injection of FCV Vaccination I in FCV-vaccinated cats. The increase in IFN-γ mRNA transcription in FCV-vaccinated cats after the first and second injection of FCV Vaccination I led to significantly higher IFN-γ levels in vaccinated cats (at day 13 P_MWU_ = 0.0317 and at day 27 P_MWU_ = 0.0317) compared to the control cats (Figure 3H). MX1 mRNA transcription levels were significantly higher in vaccinated cats compared to control cats after the first and second injection of FCV Vaccination I (at day 4 (P_MWU_ = 0.0079), at day 7 (P_MWU_ = 0.0079) and at day 22 (P_MWU_ = 0.0079)) (Figure 3G). An increase in granzyme B mRNA transcription levels was observed after the first and the second injection of FCV Vaccination I, and granzyme B was significantly higher expressed in vaccinated cats compared to control cats (at day 7 (P_MWU_ = 0.0079) and day 27 (P_MWU_ = 0.0317)) (Figure 3E). An increase in perforin mRNA transcription levels was detected after the second injection of FCV Vaccination I, and perforin levels were significantly higher in vaccinated cats than in unvaccinated ones (at day 27, P_MWU_ = 0.0317) (Figure 3D). The increase in IL-4 mRNA transcription after the second placebo injection of FCV Vaccination I in control cats led to significantly lower IL-4 levels (at day 27, P_MWU_ = 0.0317 and day 34, P_MWU_ = 0.0317) in the vaccinated cats compared to the control cats (Figure 3B). The increased Il-6 expression in the control group led to significantly lower IL-6 levels in the vaccinated group compared to the control group (day 22, P_MWU_ = 0.0317) (Figure 3K). The increase in the IFN-ω mRNA transcription level in the control cats after the first and the second placebo injection led to significantly lower IFN-ω levels in the vaccinated cats compared to the control cats (at day 4, P_MWU_ = 0.0079 and at day 20, P_MWU_ = 0.0317) (Figure 3J). The results of Friedman’s test and Dunn’s post test are shown in Table A1.

**Figure 4:**
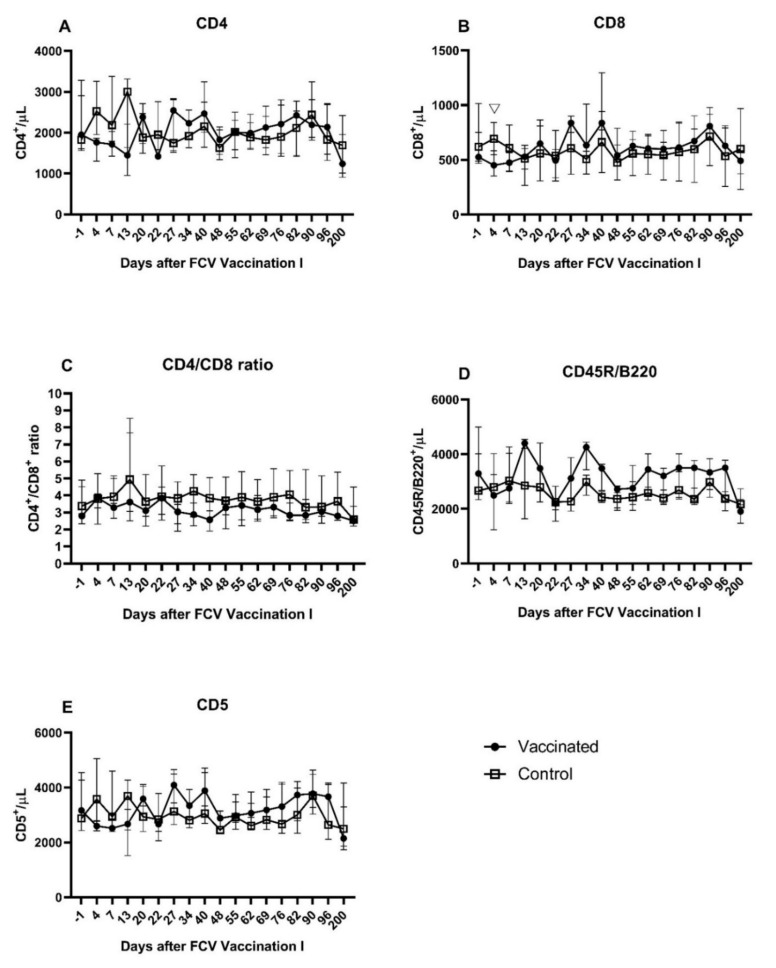
Lymphocyte subsets after FCV Vaccination I. Median (±IQR) absolute cell counts of CD4^+^ (**A**), CD8^+^ (**B**), CD4^+^/CD8^+^ ratio (**C**), CD45R/B220^+^ (**D**), and CD5^+^ (**E**) lymphocytes. Statistically significant differences between the vaccine and the control group are indicated with an open triangle; ▽ denotes significantly lower in the vaccine group P_MWU_ ≤ 0.05. The second injection of FCV Vaccination I was performed at day 21.

A transient and moderate decrease in all lymphocyte subsets was observed in FCV-vaccinated cats shortly after the first and after the second injection of FCV Vaccination I (Figure 4A–E). Both decreases were followed by a transient increase, and peak values for CD4^+^, CD8^+^, and CD5^+^ lymphocytes were reached one week after the second injection (day 27). A biphasic peak of CD45R/B220^+^ lymphocytes was observed at day 13 after the first and at day 13 after the second injection of FCV Vaccination I. A decrease in the absolute number of all lymphocyte subsets was detected at day 200 after FCV Vaccination I. Transiently lower CD8^+^ lymphocytes were detected in vaccinated cats compared to the control cats at day 4 after FCV Vaccination I (P_MWU_ = 0.0317) (Figure 4B). No further significant differences were found between the two groups in CD8^+^ lymphocyte counts and all other lymphocyte subsets (CD4^+^, CD5^+^, and CD45R/B220^+^) and the CD4^+^/CD8^+^ ratio. Significant changes over time within one group are described in Table A2.

**Figure 5:**
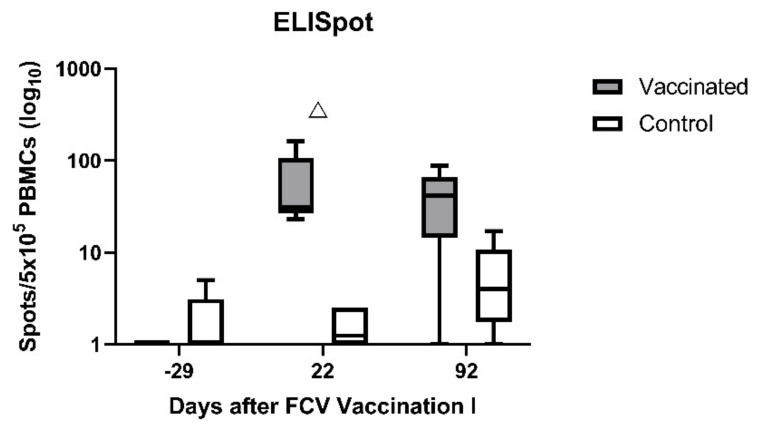
Box and whisker plots of the number of IFN-γ-releasing PBMCs after stimulation with the vaccine virus FCV F9. The line inside the box shows the median, and the lower and upper border of the box indicates the 25th and 75th percentile, respectively. Whiskers represent minimum and maximum values. Significant differences between the vaccinated group and the control are indicated with an open triangle; △ denotes significantly higher in the vaccinated group P_MWU_ ≤ 0.05. The second injection of FCV Vaccination I was performed at day 21.

Using the ELIspot assays, very few IFN-γ-releasing PBMCs were detected upon stimulation with FCV F9 in either group before FCV Vaccination I (Figure 5; day −29). After FCV Vaccination I, IFN-γ-releasing PBMCs upon stimulation with FCV F9 were detected in all FCV-vaccinated cats and only very few in the control cats (Figure 5; day 22). The FCV-vaccinated cats had significantly more IFN-γ-releasing PBMCs than the control cats at day 22 after FCV Vaccination I (P_MWU_ = 0.0079). At day 92, in four out of five FCV-vaccinated cats, IFN-γ-releasing PBMCs upon stimulation with FCV F9 were still detected, but the difference was not significant anymore between the two groups (Figure 5; day 92). Significant changes over time in each group are shown in Table A3.

### 3.2. FCV Challenge I

**Figure 6:**
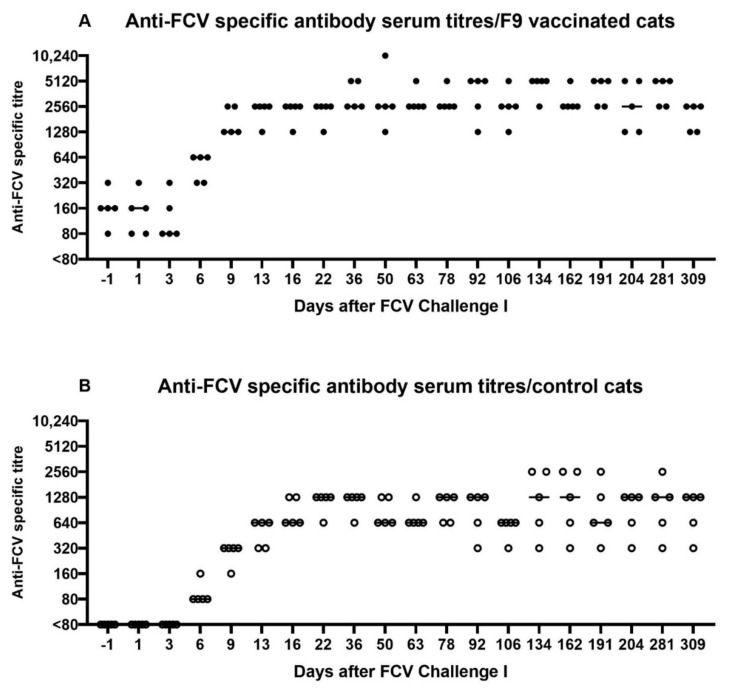
Dot plots of anti-FCV specific antibody serum titres measured by IFA before and after FCV Challenge I in the vaccinated cats (**A**) and control cats (**B**). Antibody titres reflect the reciprocal of the last serum dilution showing positive fluorescence in the IFA. Each dot represents the titre of a cat of the vaccine or control group, respectively. The horizontal bar represents the median.

All FCV-vaccinated cats had preinfectional FCV antibodies titres (80–320) as measured by IFA (Figure 6A). Titres increased in all FCV-vaccinated cats after the experimental FCV infection (FCV Challenge I), while seroconversion was detected in control cats as of day 6 on after FCV Challenge I (which corresponds to day 221 after the first injection of FCV Vaccination I) (Figure 6B). The maximum titre in the vaccine group was 10,240 and was reached at day 50 (which corresponds to day 265 after the first injection of FCV Vaccination I) by cat JJH3 (Figure 6A). In the control group, two cats (JJF1 and JJI4) reached a maximum titre of 2560 at several time points starting at day 134 (which corresponds to day 349 after the first injection of FCV Vaccination I) after FCV Challenge I (Figure 6B). The IFA antibody titres of the FCV vaccine group were on the same level or higher compared to the control cats (Figure 6A,B). The antibody titres gradually decreased in both groups until FCV Vaccination II.

**Figure 7:**
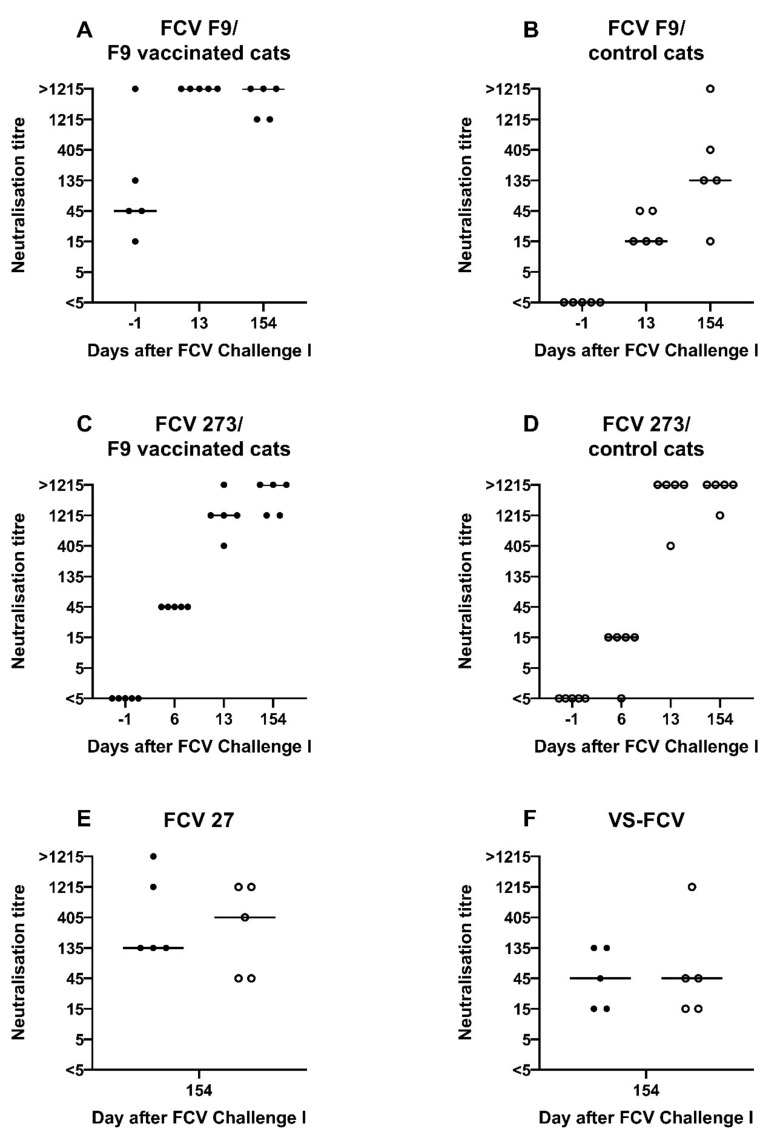
Dot plots of neutralisation titres measured by serum neutralisations assays before and after FCV Challenge I with FCV 273. Each dot represents the titre of a cat of the vaccine or control group, respectively. The titre is the reciprocal of the serum dilution where 50% or more of the wells show a CPE. Dot plot of FCV F9 neutralisation titres in vaccinated cats (**A**) and control cats (**B**), dot plot of FCV 273 neutralisation titres in vaccinated cats (**C**), and control cats (**D**), dot plot of FCV 27 neutralisation titres in vaccinated cats (solid dot) and control cats (open dot) (**E**), and dot plot of virulent-systemic (VS)-FCV-neutralisation titres in vaccinated cats (solid dot) and control cats (open dot) (**F**).

Before the first experimental infection with FCV 273 (FCV Challenge I), neutralising antibodies against FCV F9 were detected in the vaccine but not in the control group (Figure 7A,B), and no neutralising antibodies against FCV 273 were detected in either group (Figure 7C,D). At day 6 after FCV Challenge I, all five vaccinated cats and four out of five control cats had neutralising antibodies (titres 15 and 45) against the challenge virus FCV 273. One week later, the neutralisation titres against FCV 273 had increased in all cats (405, 1215, and >1215) (Figure 7C,D). For titres >1215 at day 154 after FCV Challenge I endpoint titres are shown in Table 2. Additionally, at day 154 after FCV Challenge I, the neutralisation titres against FCV F9 in the vaccinated cats were still at a high level (1215 and >1215), and the control cats had developed crossneutralising antibodies against FCV F9, reaching titres of 15 to >1215 (Figure 7A,B). All FCV 273 infected cats (FCV F9-vaccinated and control cats) had developed crossneutralising antibodies against the second challenge virus FCV 27 (prior to FCV Challenge II with FCV 27) and to a lesser degree in most cats against an unrelated Swiss VS-FCV (FCV ZH2016) field strain as determined at day 154 after FCV Challenge I (Figure 7E,F).

**Figure 8:**
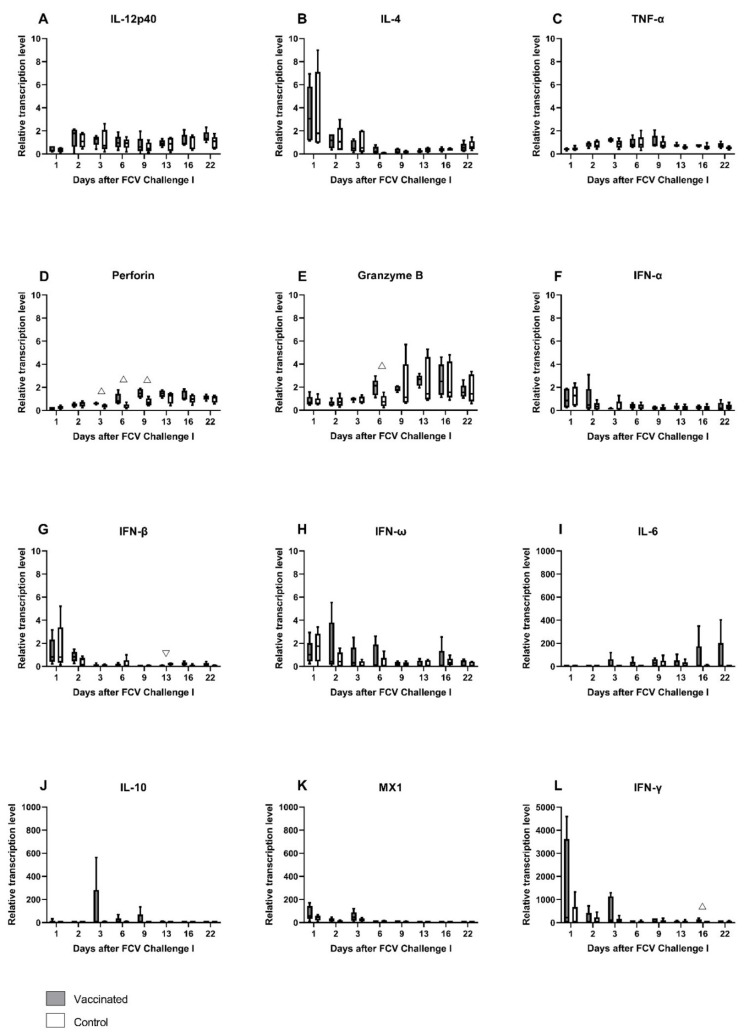
Box and whisker plots of cytokine expression profiles of IL-12p40 (**A**), IL-4 (**B**), TNF-α (**C**), perforin (**D**), granzyme B (**E**), IFN-α (**F**), IFN-β (**G**), IFN-ω (**H**), IL-6 (**I**), IL-10 (**J**), MX1 (**K**) and IFN-γ (**L**) after FCV Challenge I. The line inside the box shows the median, and the lower and upper border of the box indicates the 25th and 75th percentile, respectively. Whiskers represent minimum and maximum values. Significant differences between the vaccinated and the control group are indicated with an open triangle; ▽ denotes significantly lower in the vaccinated group P_MWU_ ≤ 0.05; △ denotes significantly higher in the vaccinated group P_MWU_ ≤ 0.05. The y-axes are scaled to either 0–10, 0–1000, or 0–5000 relative mRNA transcription levels, and the graphs are ordered, respectively.

The relative mRNA transcription levels of cytokines and proteins of the innate and the adaptive immune system that act pro- or anti-inflammatory or promote either the Th1- or Th2-differentiation of T-cells were investigated in peripheral blood (Figure 8A–L). In the early phase of FCV Challenge I, the mRNA transcription levels of IL-4, IFN-α, IFN-β, IFN-ω, MX1, and IFN-γ were transiently elevated in all cats (Figure 8B,F–H,K,L). The mRNA transcription levels of TNF-α were transiently increased in all cats from day 3 to day 9, and IL-10 was transiently increased in the FCV-vaccinated cats (Figure 8C,J). In the later phase of the infection, starting from day 6 or 9, the mRNA transcription levels of perforin and granzyme B were increased in both groups, and IL-6 mRNA transcription levels were increased predominantly in the vaccinated cats (Figure 8D,E,I). The median relative mRNA transcription level of IFN-γ when compared to pre-infection level was ≈220 times increased in the vaccinated cats at day 1 after FCV Challenge I and represents the most pronounced increase in all cytokines measured after FCV Challenge I (Figure 8L). The increase in IFN-γ mRNA transcription levels after FCV Challenge I led to significantly higher IFN-γ levels in the FCV-vaccinated cats compared to control cats (at day 16, P_MWU_ = 0.0317) (Figure 8L). Additionally, an increased IFN-β mRNA transcription was observed in cats of the control group after FCV Challenge I and led to significantly lower IFN-β values in vaccinated cats compared to control cats (at day 13, P_MWU_ = 0.0079) (Figure 8G). Perforin and granzyme B mRNA transcription levels were increased in vaccinated cats after FCV Challenge I and led to significantly higher levels in vaccinated cats compared to control cats for perforin (at day 3, P_MWU_ = 0.0159; day 6, P_MWU_ = 0.0317; and day 9, P_MWU_ = 0.0317) and for granzyme B (at day 6, P_MWU_ = 0.0317) (Figure 8D,E). Significant changes in cytokine mRNA transcription levels in each group over time are shown in Table A4.

**Figure 9:**
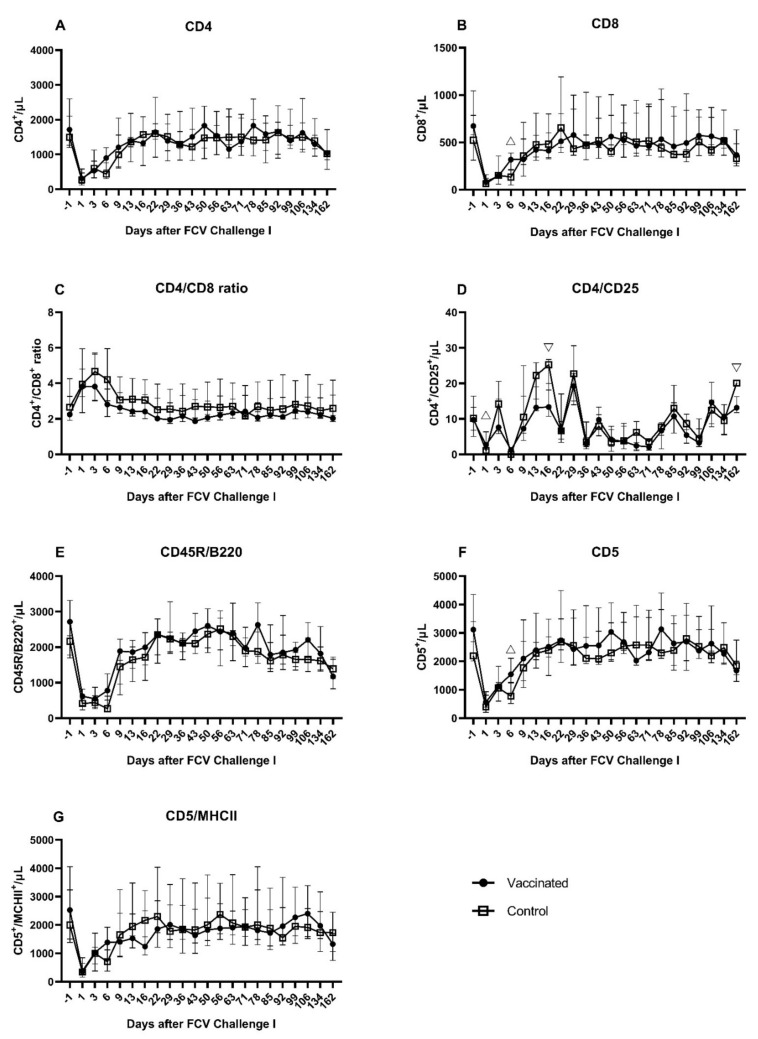
T- and B-lymphocytes subsets after FCV Challenge I. Median (±IQR) absolute cell counts of CD4^+^ (**A**), CD8^+^ (**B**), CD4^+^/CD8^+^ ratio (**C**), CD4^+^/CD25^+^ (**D**), B-lymphocytes (CD45R/B220^+^) (**E**), CD5^+^ (**F**), and CD5^+^/MHCII^+^ (**G**). Statistically significant differences between the vaccine and the control group are indicated with open triangles; ▽ denotes significantly lower in the vaccinated group P_MWU_ ≤ 0.05; △ denotes significantly higher in the vaccinated group P_MWU_ ≤ 0.05.

During FCV Challenge I, T-cells (CD5^+^) activated T-cells (CD5^+^/MHCII^+^), B-cells (CD45R/B220^+^), T-helper cells (CD4^+^), T regulatory cells (CD4^+^/CD25^+^), and cytotoxic T-cells (CD8^+^) were measured by flowcytometry (Figure 9A–G). The lymphocyte subsets (absolute counts) of CD5^+^, CD5^+^/MHCII^+^, and CD4^+^ were significantly decreased at day 1 after FCV Challenge I in both groups compared to preinfectional levels (Table A5). The lymphocyte subsets (absolute counts) of CD45R/B220^+^ and CD8^+^ were significantly decreased at day 1 after FCV Challenge I in the vaccinated cats compared to preinfectional levels (Table A5). The CD4^+^/CD8^+^ ratio transiently increased at day 1 and day 3 after FCV Challenge I and decreased thereafter and returned to preinfectional level around day 22 in both groups (Figure 9C). All subsets except CD4^+^/CD25^+^ returned to (or were above) the preinfectional level by day 22 post infection (Figure 9A–G). CD4^+^/CD25^+^ cells were significantly higher in the vaccine group than in the control group at day 1 (P_MWU_ = 0.0317) and significantly lower in the vaccine group at days 16 (P_MWU_ = 0.0317) and 162 (P_MWU_ = 0.0079) after FCV Challenge I (Figure 9D). CD8^+^ cells were significantly higher in the vaccine group than in the control group at day 6 after FCV Challenge I (P_MWU_ = 0.0317) (Figure 9B). CD5^+^ lymphocytes were significantly higher in the vaccinated cats than in the control cats at day 6 after FCV Challenge I (P_MWU_ = 0.0317) (Figure 9F).

**Figure 10:**
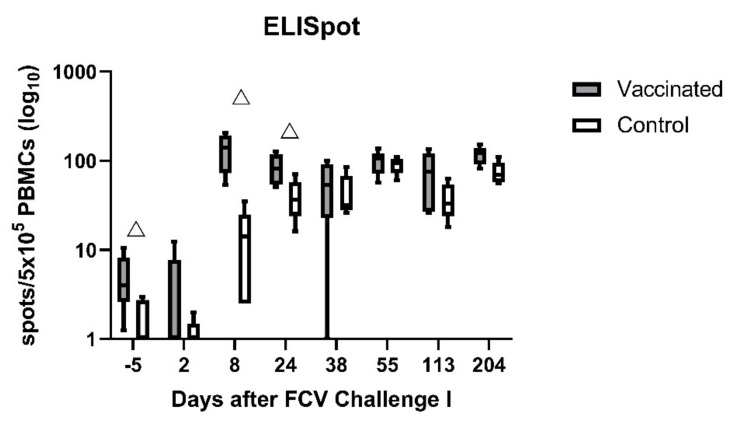
Box and whisker plots of IFN-γ-releasing PBMCs after stimulation with the challenge virus FCV 273. The line inside the box shows the median, and the lower and upper border of the box indicates the 25th and 75th percentile, respectively. Whiskers represent minimum and maximum values. Significant differences between the vaccinated and the control group are indicated with an open triangle; △ denotes significantly higher in the vaccinated group P_MWU_ ≤ 0.05.

The FCV-vaccinated cats had significantly higher values of detectable INF-γ-releasing PBMCs stimulated with FCV 273 before FCV Challenge I (day −5; P_MWU_ = 0.0238) than control cats. The vaccinated cats reached the highest value at day 8 after FCV Challenge I (Figure 10). INF-γ-releasing PBMCs were significantly higher in FCV-vaccinated cats at days 8 (P_MWU_ = 0.0159) and 24 (P_MWU_ = 0.0317) after FCV Challenge I. No spot number could be obtained for cat JJI2 of the vaccine group at day 8 after FCV Challenge I due to insufficient sample material. The control cats started to have IFN-γ-releasing PBMCs from day 8 after FCV Challenge I on and reached levels not significantly different anymore from FCV-vaccinated cats from day 38 after FCV Challenge I on. IFN-γ-releasing PBMCs upon stimulation with FCV 273 were detectable in both groups until day 204 after FCV Challenge I. Significant changes over time are presented in Table A6.

### 3.3. FCV Vaccination II

**Figure 11:**
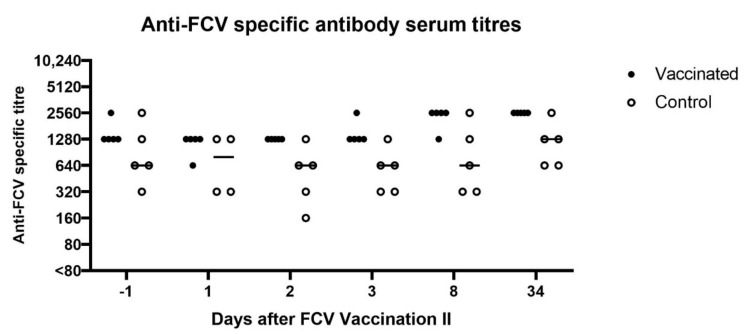
Dot plot of anti-FCV specific antibody serum titres measured by IFA before and after FCV Vaccination II in vaccinated and control cats, respectively. Antibody titres reflect the reciprocal of the last serum dilution showing positive fluorescence in the IFA. Each dot represents the titre of a cat of the vaccine or control group, respectively. At day 1, no IFA titre could be measured in the cat JJI4 of the control group due to not enough sample material. The horizontal bar represents the median. Day 34 correspond to day −1 of FCV Challenge II.

All cats were revaccinated (FCV F9) or placebo injected, 336 days after FCV Challenge I with FCV 273. The cats in the control group had similarly high seroreactive titres measured by IFA as the FCV-vaccinated cats (both groups between 320 and 2560) pre-revaccination (day −1 of FCV Vaccination II), and the IFA FCV titres also stayed high in both groups after vaccination with some fluctuation (Figure 11).

**Figure 12:**
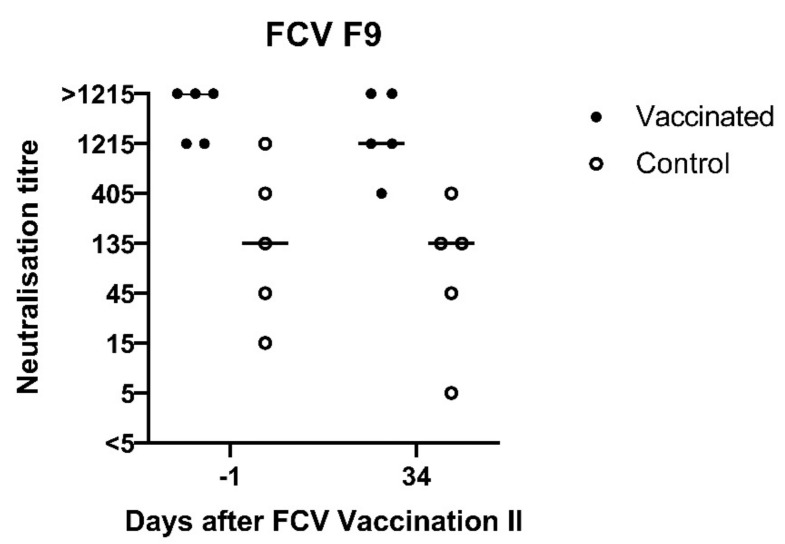
Dot plot of FCV F9 neutralisation titres measured by serum neutralisations assays before and after FCV Vaccination II with FCV F9. The titre is the reciprocal of the serum dilution where 50% or more of the wells show a CPE. Each dot represents the titre of a cat of the vaccine or control group, respectively. The horizontal bar represents the median. Day 34 correspond to day −1 of FCV Challenge II.

At day −1 before FCV Vaccination II, the neutralising antibodies against FCV 273 in all cats (titres 405, 1215 and >1215) and against FCV F9 in the vaccinated group were still at a high level (1215 and ≥1250, Figure 12), while the neutralising antibody titres against FCV F9 in the control group were lower, with four out of five cats having titres <1215 (Figure 12). One month after FCV Vaccination II, the titres against FCV F9 stayed at the same level in three out of five FCV F9-vaccinated cats and dropped one titre step in two out of five vaccinated cats but were still higher in four out of five cats of the vaccine group (titres >405) compared to the control group (titres 5–405).

### 3.4. FCV Challenge II

**Figure 13:**
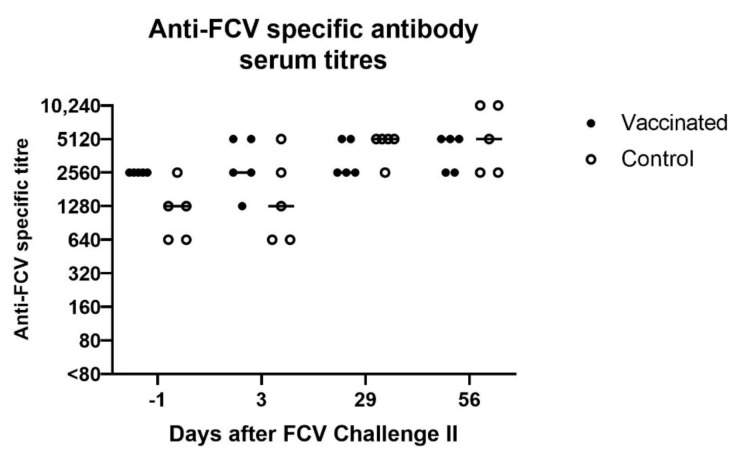
Dot plot of anti-FCV specific antibody serum titres measured by IFA before and after FCV Challenge II in vaccinated and control cats, respectively. Antibody titres reflect the reciprocal of the last serum dilution showing positive fluorescence in the IFA. Each dot represents the titre of a cat of the vaccine or control group, respectively. The horizontal bar represents the median.

At day 3 after FCV Challenge II (corresponds to day 38 after FCV Vaccination II), in two out of five cats in the vaccine and the control group, respectively, increased FCV IFA titres were detected, while in the other three cats of each group either the same titre or a lower titre was detected compared to day −1 of FCV Challenge II (corresponds to day 34 after FCV Vaccination II) (Figure 13). Over the 56 days observation period of FCV Challenge II, a titre increase was observed in three out of five cats of the vaccine group and in all cats of the control group.

**Figure 14:**
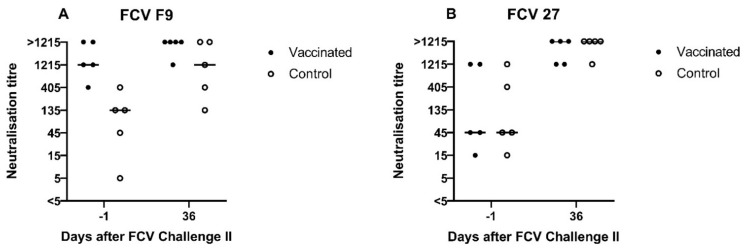
Dot plots of FCV F9 (**A**) and FCV 27 (**B**) neutralisation titres measured by serum neutralisations assays before and after FCV Challenge II with FCV 27. Each dot represents the titre of a cat of the vaccine or control group, respectively. The titre is the reciprocal of the serum dilution where 50% or more of the wells show a CPE. The horizontal bar represents the median.

A low neutralising antibody titre against FCV F9 of 5 was detected in cat JJG3 of the control group before FCV Challenge II, whereas titres of 45 or higher were observed in all nine other cats (Figure 14A). Neutralising antibodies against FCV 27 were already present in both groups before FCV Challenge II with FCV 27 (Figure 14B). The neutralising titres against FCV 27 ranged from 15 to 1215 in all cats before FCV Challenge II. At day 36 after FCV Challenge II (corresponds to day 71 after FCV Vaccination II), neutralising antibody titres of 1215 or >1215 against FCV 27 and titres of 135 to >1215 against FCV F9 were detected in all cats (Figure 14A,B). Endpoint tires against FCV 27 are shown in Table 3.

**Figure 15:**
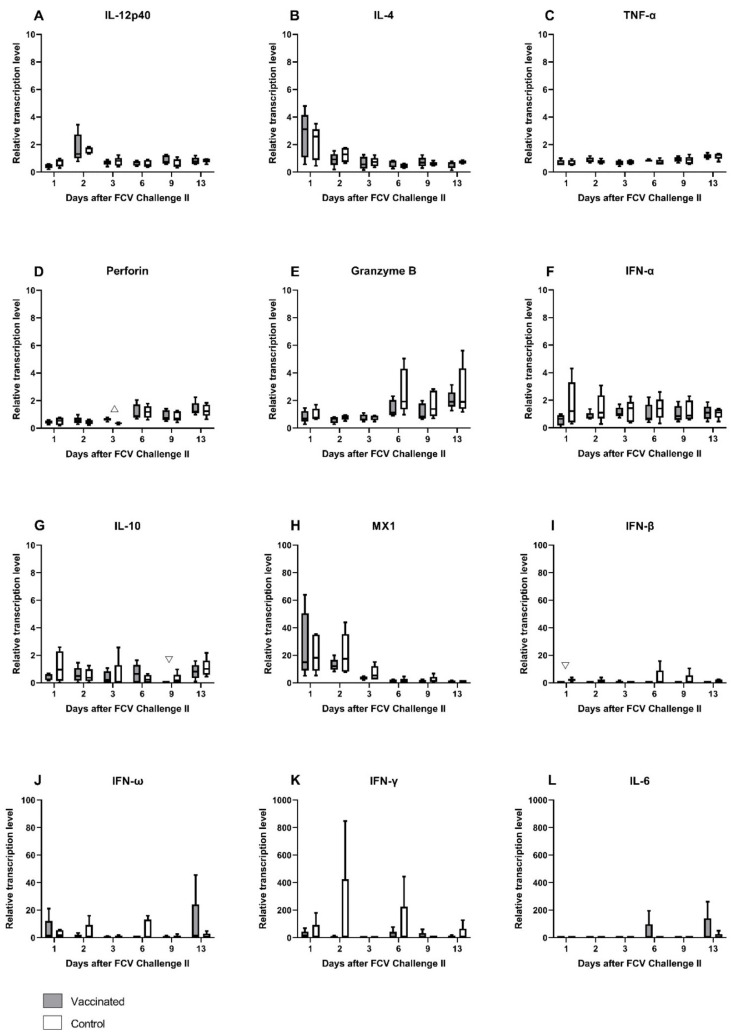
Box and whisker plots of cytokine expression profiles of IL-12p40 (**A**), IL-4 (**B**), TNF-α (**C**), perforin (**D**), granzyme B (**E**), IFN-α (**F**), IL-10 (**G**), MX1 (**H**), IFN-β (**I**), IFN-ω (**J**), IFN-γ (**K**) and IL-6 (**L**) after FCV Challenge II. The line inside the box shows the median, and the lower and upper border of the box indicates the 25th and 75th percentile, respectively. Whiskers represent minimum and maximum values. Significant differences between the vaccinated and the control group are indicated by open triangles; △ denotes significantly higher in the vaccine group P_MWU_ ≤ 0.05; ▽ denotes significantly lower in the vaccine group P_MWU_ ≤ 0.05. The y-axes are scaled to either 0–10, 0–100, or 0–1000 relative mRNA transcription levels, and the graphs are ordered, respectively.

The relative mRNA transcription levels of cytokines and proteins of the innate and the adaptive immune system that act pro- or anti-inflammatory or promote either the Th1- or Th2-differentiation of T-cells were investigated in peripheral blood (Figure 15A–L). As after FCV Challenge I, the median mRNA transcription levels of IL-12p40 (day 2), IL-4 (day 1), and MX1 (mainly day 1 and day 2) were transiently increased, whereas perforin was decreased in the early phase of the infection (Figure 15A,B,D,H) in both groups. mRNA transcription levels of perforin and granzyme B (from day 6 on) were increased in the later phase of the infection in both groups (Figure 15D,E). Furthermore, in the later phase of FCV Challenge II, the mRNA transcription levels of IL-6 and IFN-ω were predominantly increased in the FCV-vaccinated cats, whereas IFN-β mRNA transcription levels were upregulated in the control cats (Figure 15I,J,L). INF-γ was predominantly upregulated in the control cats over the whole observation period of FCV Challenge II (Figure 15K). Significantly higher levels of IL-10 were observed in the control group at day 9 (P_MWU_ = 0.0317) compared to the vaccine group (Figure 15G). IFN-β was significantly lower in the vaccinated cats at day 1 compared to the unvaccinated cats (P_MWU_ = 0.0079) (Figure 15I). No statistically significant differences were detected for IFN-α and IFN-ω (Figure 15F,J). At day 3, perforin levels were significantly higher in the vaccinated cats than in the unvaccinated cats (P_MWU_ = 0.0079) (Figure 15D). No clear tendency of up- or down-regulation could be observed for TNF-α, IFN-α, and IL-10 (Figure 15C,F,G). The relative mRNA transcription level of IFN-γ was statistically not significantly different between the groups (Figure 15K). Looking at the individual cats, JJI3 from the control group had an increase in IFN-γ relative mRNA transcription level of up to 840 times, and cat JJI2, from the vaccine group, had an increase of up to 60 times compared to preinfectional levels. Statistically significant results for changes over time in each group are presented in Table A7.

**Figure 16:**
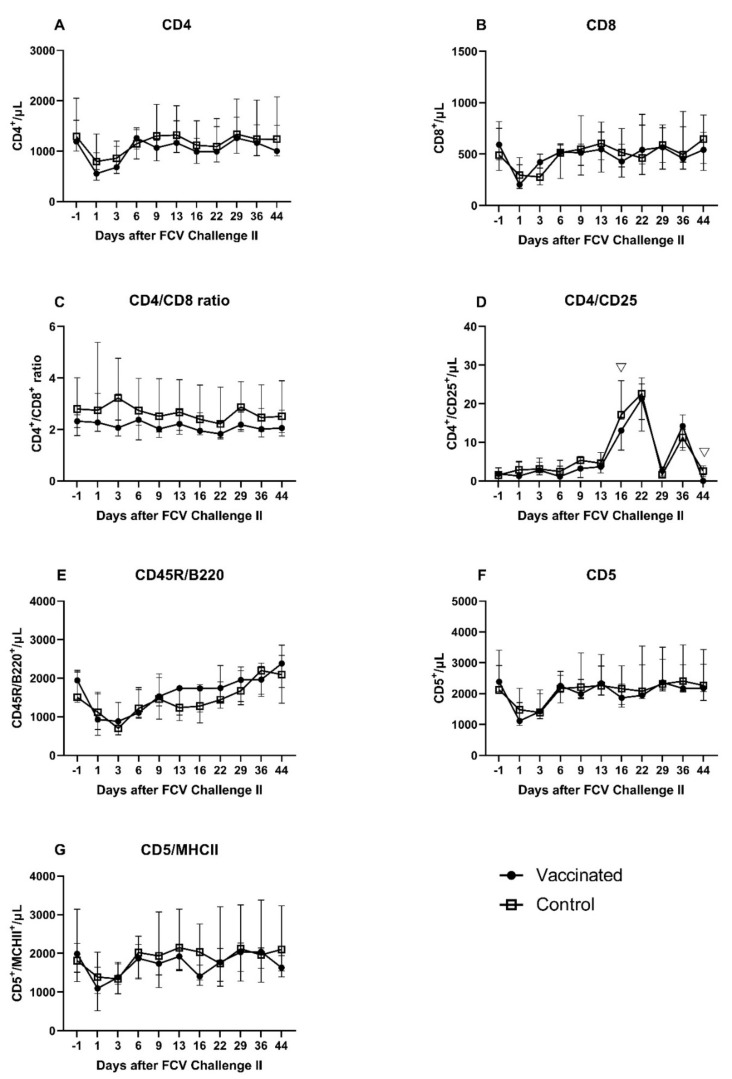
T- and B-lymphocyte subsets after FCV Challenge II. Median (±IQR) absolute cell counts of CD4^+^ (**A**), CD8^+^ (**B**), CD4^+^/CD8^+^ ratio (**C**), CD4^+^/CD25^+^ (**D**), B lymphocytes (CD45R/B220^+^) (**E**), CD5^+^ (**F**), and CD5^+^/MHCII^+^ (**G**) lymphocytes. Statistically significant differences between the vaccine and the control group are indicated with open triangles; ▽ denotes significantly lower in the vaccinated group P_MWU_ ≤ 0.05.

As in after the FCV Challenge I but to a lesser extent the lymphocyte subsets of CD4^+^, CD8^+^, CD45R/B220^+^, CD5^+^, and CD5^+^/MHCII^+^ showed a transient decrease in absolute cell numbers at day 1 and day 3 after FCV Challenge II (Figure 16A–G). Similar to post FCV Challenge I, three peak values were observed at day 16, day 22, and day 36 after FCV Challenge II for CD4^+^/CD25^+^ lymphocytes in both groups (Figure 16D). Contrary to FCV Challenge I, CD4^+^/CD25^+^ lymphocytes stayed at the preinfectional level in the early phase of FCV Challenge II. CD4^+^/CD25^+^ lymphocytes were significantly lower in the FCV-vaccinated cats than in the control cats at day 16 (P_MWU_ = 0.0079) and day 44 (P_MWU_ = 0.0317) (Figure 16D). As in after the FCV Challenge I but to a lower extent and only in the control group, the CD4^+^/CD8^+^ ratio increased shortly after FCV Challenge II, and the ratio returned to the preinfectional level by day 6 (Figure 16C). Significant changes over time in each group are presented in Table A8.

**Figure 17:**
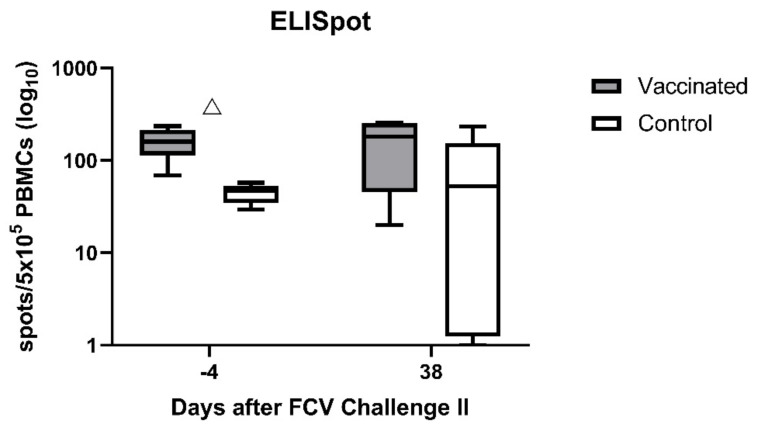
Box and whisker plots of IFN-γ-releasing PBMCs after stimulation with the challenge virus FCV 27. The line inside the box shows the median, and the lower and upper border of the box indicates the 25th and 75th percentile, respectively. Whiskers represent minimum and maximum values. Significant differences between the vaccinated and the control group are indicated with an open triangle; △ denotes significantly higher in the vaccinated group P_MWU_ ≤ 0.05.

Prior to FCV Challenge II (day −4), INF-γ-releasing PBMCs were detectable in both groups (FCV-vaccinated and controls) upon stimulation with FCV 27 to some degree; however, FCV-vaccinated cats had a significantly higher number of IFN-γ-releasing PBMCs than control cats (P_MWU_ = 0.0079; Figure 17). After FCV Challenge II (using FCV 27), the median number of IFN-γ-releasing PBMCs upon stimulation with FCV 27 was somewhat higher in the control group, and no significant difference was observed anymore between the two groups. No statistically significant changes over time were detected in either group (Table A9).

## 4. Discussion

Antibodies against FCV are considered pivotal for protection against FCV-induced disease; at the same time, cats without detectable antibodies can also be protected from disease [17,19,20]. The latter indicates that for protection from FCV-induced diseases, innate and adaptive cell-mediated immunity are also of importance. Nonetheless, very little is known about the cell-mediated immune response after FCV modified-live vaccination and heterologous FCV challenge infection. This knowledge gap is addressed in the present study where SPF cats were vaccinated subcutaneously against FCV with a modified-live vaccine containing FCV F9 or with a placebo vaccine and subsequently challenged with heterologous FCV field isolates. Innate and adaptive humoral and cellular immune mechanisms were evaluated by detection of neutralising antibody responses, cytokine mRNA transcription levels, lymphocyte subsets, and INF-γ-releasing PBMCs after FCV vaccinations and challenge infections. The initial FCV vaccination (FCV Vaccination I) evoked a cellular and humoral immune response against the vaccine strain FCV F9 as well as a cellular but not a humoral crossreactive immunity against the first FCV challenge virus (FCV 273) and a Th1-directed cytokine response in vaccinated cats. Furthermore, innate immune mechanisms, such as antiviral factor MX1 and perforin and granzyme B mRNA transcription levels, were elevated in vaccinated cats compared to control cats after vaccination with the modified-live FCV F9 vaccine. The data describing the clinical manifestations of FCV infection in the vaccinated and the control cats as well as the FCV shedding, the FCV RNAemia, the FCV loads, the haematological changes, and the inflammation have been described previously [28].

After the first challenge with a current FCV field strain (FCV 273; FCV Challenge I), an adaptive immune response was detected in vaccinated cats with higher absolute numbers of T-cells (CD5), cytotoxic T-cells (CD8) and regulatory T-cells (CD4/CD25), and higher mRNA transcription levels of perforin and granzyme B compared to placebo-vaccinated and challenged control cats. Of note, after FCV Challenge I, the vaccinated cats showed less severe clinical signs, shed less FCV RNA from the oropharynx, and FCV RNA in blood was detected over a shorter duration compared to the control cats [28]. The first experimental infection with FCV 273 induced humoral and cellular crossimmunity against the second challenge virus, the field strain FCV 27, in all cats, but vaccinated cats still had more IFN-γ-releasing PBMCs upon stimulation with FCV 27 before FCV Challenge II with FCV 27 compared to placebo-vaccinated control cats. FCV Challenge II with FCV 27 caused only mild changes in lymphocyte subsets, cytokine mRNA transcription levels and antibody levels in all cats, and fewer timepoints were significantly different between the groups. The results of the clinical presentation, FCV shedding, RNAemia, haematology, and acute phase protein response in the cats of this study have been described previously [28]. Of note, after FCV Challenge II, no clinical signs and no FCV RNA in blood were detected in any of the cats [28].

In the present study, antibodies against FCV were measured by IFA and by neutralisation assays. Virus neutralisation assays are labour intensive and require specialised laboratory settings and equipment. In IFA assays, no handling of infectious FCV is needed once the slides are prepared. However, IFA detects the binding of antibodies to the antigen and not the biological function. To assess the biological function, virus neutralisation assays were performed in parallel in the present study. The exact titre that correlates with protection is not conclusively known for either assay, IFA or neutralisation. In the neutralisation study of Povey and Ingersoll 1975 [16], it was suggested that a titre of 1:16 or greater indicates protection, and a titre of 1:7 or lower indicates susceptibility to a challenge with heterologous FCV strains. Therefore, in the present study, neutralisation titres >7 were considered to be neutralising. Another study indicated that in vaccinated cats, the detection of any FCV-specific antibodies determined by ELISA or virus neutralisation was predictive for protection against disease independent of the titre; however, this study used different vaccine and challenge viruses than the present study [42], and the results might not be directly comparable.

The first injection of the modified-live vaccine containing FCV F9 induced antibodies measured by IFA and by neutralisation assays in all vaccinated cats against the vaccine strain F9. The onset of detectable antibodies by day 13 was similar to that reported in previous studies [43,44,45] and coincided with a peak in B lymphocyte numbers (CD45R/B220). A single injection with the modified-live vaccine resulted in neutralising antibodies against the vaccine strain, while no crossneutralising antibodies against the heterologous FCV 273 could be detected at this time. The second injection of FCV Vaccination I caused an increase in IFA titres in all five vaccinated cats and an increase in neutralisation titre in two of the five vaccinated cats.

In vaccine recommendations for cats, two to three vaccinations 3–4 weeks apart starting around 8 weeks of age are recommended to induce a reliable immune response [8,46]. These recommendations are based on findings from the field where maternal antibodies in kittens can interfere with the development of vaccine immunity [47]. The SPF cats used in the present study were free of maternal FCV antibodies, since the queens had not been vaccinated and the cattery was free of FCV. Under these circumstances, a neutralising immune response was induced after one vaccination. This finding is in line with a previous report where FCV-neutralising antibodies were found in SPF cats from day 14 onwards after one subcutaneous administration of a MLV vaccine containing FCV, feline panleukopenia virus, and feline herpesvirus [45]. These findings should be taken into consideration for vaccination decisions in adult unvaccinated cats where two vaccinations, from three to four weeks apart, are not feasible, e.g., due to cost restrictions or the inability to recapture feral cats. In these cases, one injection of a MLV FCV vaccine might be sufficient to induce some immunity in a previously naïve adult cat.

The homologous neutralisation titres against FCV F9 detected after FCV Vaccination I in the current study were generally lower compared to those reported in cats that were experimentally infected with high doses of the vaccine strain F9 [22]. It is known that vaccination usually does not trigger an identical immune response as an infection, but differences should be minimal if MLV vaccines are used [48]. The artificial attenuation of the virus, the subcutaneous route of infection, and the potential lower viral titre in the MLV vaccine represent the main differences compared to an acquired FCV infection. The serum of one cat in the present study, cat JJI2, showed the same neutralisation titre (1215) after one injection as a cat serum that was obtained through infection of a SPF cat with FCV F9 in a previously reported study [22]. The latter serum was used in the study of Addie et al. (2008) [22], to test field isolates for susceptibility to neutralisation by antiserum raised against F9. It was thought that through vaccination alone, it is impossible to reach a serum titre that has a sufficient homologous neutralisation ability [18,23]. Our finding confirms and extends the recent studies of Afonso et al. (2017) [49] and Smith et al. (2019) [24], where anti-F9 serum samples from hyperimmunised cats (20 immunisations) were used for FCV neutralisation. For further neutralisation studies of FCV field isolates, the use of anti-F9 serum obtained through vaccination should be favoured, and hyperimmunisation might not even be needed. Infections, in contrast to vaccinations, possibly activate additional immune mechanisms [23], and antibodies elicited through a licensed vaccination protocol would mirror more precisely the field situation in vaccinated cats. Interestingly, only one out of five vaccinated cats (cat JJI2) developed a high neutralisation titre of 1215 after the first injection of Vaccination I. The other four cats had neutralisation titres of 135 (JJG4 and JJH3) and 45 (JJG6, JJI1). The same vaccination procedure in SPF cats of the same age, and some cats of the same litter (JJI1 and JJI2) and sex living in identical housing conditions, had different outcomes regarding the magnitude of the humoral immune reaction. The same was observed in the study of Addie et al. (2008) [22], with the infection of SPF cats with different FCV vaccine strains, and in the study of Afonso et al. (2017) [49] with hyperimmunisation of SPF cats through repeated vaccinations. They state that the differences in titres observed in different cats under the same circumstances could be due to nonspecific immune mechanisms that have not been investigated thus far in cats. Therefore, both the virus characteristics and the host immune response influence the success of vaccination or the outcome of an FCV infection. Factors such as co-infections, immunosuppression through medications or infections, age, and housing conditions can be ruled out in the present study. This points to genetic or stress-related factors, such as high cortisol levels in some cats due to group housing or brief adrenaline/noradrenaline release during the process of cat handling and vaccine injection. The cats in the present study were trained and generally well adapted to the procedures but individual differences in the excitement level cannot be ruled out completely. Interestingly, the cat with the neutralisation titre of 1215 (JJI2) developed only very mild clinical signs, and no RNAemia was detected after FCV Challenge I; the cat did not shed FCV after FCV Challenge II [28]. The other four vaccinated cats had also significantly lower clinical scores than the control cats, but RNAemia was detected after FCV Challenge I, and FCV was shed after FCV Challenge II. Further investigations regarding non-specific immune mechanisms need to be undertaken in the future to improve the immune response towards the currently available FCV vaccines.

The FCV F9 vaccination induced detectable crossneutralising antibodies neither against the first (FCV 273) nor the second challenge virus (FCV 27). This finding is in concordance with the neutralisation results that were obtained previously for challenge virus 273 [28], where no neutralisation against FCV F9 was detected (titres <5 and 5). However, the absence of crossneutralisation of FCV 27 by serum samples of FCV F9-vaccinated cats does not correspond with our previous finding that FCV 27 can be neutralised by serum samples of FCV-F9-infected cats with a titre of 15 [28]. This confirms that the antibody response obtained through infection is not identical to that elicited through vaccination and indicates that studies investigating neutralisation of different FCV strains should not use sera from infected cats but rather from vaccinated cats. Additionally, no crossneutralisation was detected against the Swiss VS-FCV (FCV ZH2016), which was isolated from a severe outbreak of virulent systemic disease in the small animal hospital of the University of Zurich at the end of 2016, which necessitated a closure of the clinic for three weeks (personal communication A.M.S., F.S.B., and R.H.L.). This finding is in line with most reports about VS-FCV indicating no protective effect of FCV vaccination against VS-FCV induced disease [9,11,15]. However, the crossprotection against FCV-induced disease in the absence or with low levels of heterologous antibodies has been shown previously [17,18,19,20], and cell-mediated immune mechanisms also play a role in vaccine immunity. The unvaccinated cats did not develop antibodies against F9, which confirms our finding that the vaccine virus was not shed in an amount that was detectable by RT-PCR [28], ruling out the potential for inadvertent immunisation of the unvaccinated cats.

The first experimental infection with FCV 273 induced crossneutralising antibodies in all cats against FCV F9, FCV 27, and a Swiss VS-FCV (FCV ZH2016) isolate. The development of neutralising antibodies against the challenge virus started as early as six days after the FCV Challenge I; by that time, 9/10 cats already had neutralising antibodies. A similar pattern was observed for antibodies measured by IFA. The challenge virus FCV 273 seems to be very efficient in stimulating a broad humoral immune response and should therefore be tested in the future for its neutralisation ability against other FCV strains.

FCV Vaccination II, which was performed 11 months after FCV Challenge I, did not induce marked changes in the IFA and neutralisation titres. The increase or decrease of one titre step most probably reflects biological and interassay variation. High prevaccination antibody titres might have hampered an increase in vaccine-induced antibodies, but a recent study found no association between high prevaccination FCV antibody titre and a lower humoral response after FCV vaccination [50]. A delay in the induction of protective antibodies has been described for kittens with high levels of maternally derived antibodies [47], and cats with high antibody titres against feline panleukopenia virus were found to react less to a subsequent feline panleukopenia vaccination [51]. Interestingly, for FCV, a titre decrease has been described in some cats with pre-existing antibodies after FCV vaccination [50]. Even though no massive titre increase was observed in the present study, the immunological memory might have been ameliorated by an increase in the quality of the humoral immune response through an increased binding capacity and higher affinity of the antibodies.

Similar to the FCV Challenge I, the FCV Challenge II induced an increase in IFA and neutralisation titres against FCV 27 and FCV F9 in all cats that had preinfectional titres of <2560 in IFA and <1215 in neutralisation. This finding implies that stimulation of the immune system with a second heterologous FCV strain increased the magnitude of crossneutralising antibodies. After both experimental FCV infections no indications implying original antigenic sin (OAS) were observed. OAS is a concept in immunology where the immune system relies on the memory obtained through a previous infection. If a subsequent infection with the same pathogen but with slightly modified epitopes occurs, the immune system does not adapt to the new epitopes and is frozen in its original repertoire of immunological defence [52]. The concept of OAS has been shown to interfere with vaccination success for various pathogens such as zika virus [52], dengue virus [52], influenza virus [52], or human norovirus [53], and OAS should be considered when developing new vaccines. However, as for FCV, no indications of OAS were observed, and the development of new vaccines including several FCV strains, even given consecutively, should be evaluated in the future.

This study presents for the first time a comprehensive description of cytokine mRNA transcription levels in the blood of FCV-vaccinated and/or FCV-challenged SPF cats. The relative mRNA transcription levels of cytokines and proteins of the innate and the adaptive immune system that act pro- or anti-inflammatory or promote either the Th1- or Th2-differentiation of T-cells were investigated. Cytokines IL-12p40, IFN-α, IFN-β, IFN-ω, IL-6, IL-10, and TNF-α represent mainly cytokines of the innate immune system, while IL-4 and IFN-γ represent mainly cytokines of the adaptive immune system, and all of them are involved in the bridging between the innate and adaptive immune systems [54]. Furthermore, the mRNA transcription levels of effector proteins such as perforin, granzyme B, and antiviral factor MX1 were investigated. The overall cytokine response after FCV Vaccination I indicated an activation of the T-helper cell type (Th1 lineage) with higher expression values in vaccinated cats than in control cats for IFN-γ and lower expression values for IL-4 and IL-6. T-helper cells type 1 have an important role in the adaptive cell-mediated immune response against intracellular pathogens, such as viruses, by activating cytotoxic T-cells [55]. In addition to the adaptive immune response, mechanisms of the innate immune system were also activated. In vaccinated cats, granzyme B levels were elevated one week after the first injection and likewise for both granzyme B and perforin levels after the second injection of FCV Vaccination I. Perforin and granzyme B are cytotoxic effector molecules stored in granules and are a part of the innate immunity [54]. Perforin and granzyme B act cooperatively upon stimulation with IFN-γ from natural killer (NK) cells and CD8^+^ cytotoxic T-cells in activating apoptotic pathways in virus-infected cells [32,56]. Cells without an MHCI receptor are the main targets of NK cells. Viral pathogens develop mechanisms to downregulate MHCI expression of their host cells with the aim of avoiding antigen presentation on MHCI, and therefore, effector T-cells are not able to recognise the infected cells [57]. NK cells rapidly detect MHCI receptor free cells and immediately induce apoptosis. Cell death by apoptosis is the preferred way of eliminating virus-infected cells. During apoptosis, the virus particles in the cytoplasm of a cell are not uncontrollably released, and no unwanted inflammatory response due to spilled cell material attracting lymphocytes is elicited [57]. Cell death by necroptosis can be preferred by cytolytic viruses as this helps in the spreading of viral particles into nearby cells [58]. The first and the second injection of FCV Vaccination I activated the apoptotic pathway of the innate immune system represented by perforin and granzyme B.

Additionally, the mRNA of the myxovirus resistance gene 1 (MX1) was significantly higher expressed in the vaccinated cats than in the control cats immediately after the first and the second injection of FCV Vaccination I. MX1 is a dynamin-like GTPase antiviral protein and an important mediator in antiviral defence by restricting viral replication [59,60]. The antiviral role of MX1 is well studied in human negative-strand RNA virus infections such as influenza [59,61]. In a study of Robert-Tissot et al. (2012) [62], upregulation of MX1 mRNA expression was correlated with FCV inhibition in vitro, indicating an antiviral effect of MX1 against FCV. MX1 is induced by type I and type III IFNs. Therefore, it would be expected that if MX1 mRNA transcription is upregulated in vaccinated cats, type I IFN as IFN-α, IFN-β, and IFN-ω mRNA transcription levels would also be elevated. For IFN-α, an upregulation in vaccinated cats was seen at day 7 after vaccination, but the difference was not statistically significant between the groups, while for IFN-β and IFN-ω, an upregulation was seen in the control cats but not in the vaccinated cats, and the difference was statistically significant for IFN-ω at days 4 and 20 after vaccination. Tian et al. (2015) [63], found that live FCV F9 did not activate the IFN-β promoter, and thus IFN-β mRNA transcription could not start, and therefore F9 might have the ability to evade the host INF-β response. As a result of this, the attenuated FCV F9 vaccine virus may also evade not only the INF-β but also the IFN-ω host immune response.

After FCV Challenge I, activation of the early innate immune system was detected in all cats independently of vaccination status, with increased mRNA transcription levels of IFN-γ, IFN-α, IFN-β, IFN-ω, and antiviral factor MX1 compared to preinfectional level. These cytokines, especially type I IFN such as IFN-α, IFN-β and IFN-ω, are important mediators of the early innate immune response to viral infections, and in the present study these mediators were activated independent of vaccination status. High IFN-γ mRNA transcription levels were observed from day 1 to day 3 after FCV Challenge I in cats of both groups. At day 16, IFN-γ mRNA transcription levels were significantly higher in vaccinated than in control cats. IFN-γ is produced mainly by NK cells, T-helper-cells, and cytotoxic T-cells [64], and it activates macrophages, helps in the differentiation of naïve CD4^+^ T-cells into the Th1 lineage, and inhibits the differentiation of Th2-cells [54]. Additionally, IFN-γ stimulates the release of perforin and granzyme B that are stored in granules of NK cells and cytotoxic T-cells. In the present study, perforin and granzyme B were faster and more intensely activated in the vaccinated cats than in the control cats. The cytokine mRNA transcription pattern seen after FCV infection indicates a more pronounced response of NK cells, cytotoxic cells, and an activation of the cytolytic and apoptotic pathway in vaccinated compared to control cats. IFN-β was significantly lower at day 13 in the vaccinated cats compared to the control cats, but the mRNA transcription levels were very low (<1) in both groups, and this difference is most probably not biologically relevant. The expression of cytokines could have been influenced by the short-time non-steroidal anti-inflammatory treatment with meloxicam of three cats in the control group. This treatment was necessary for animal welfare as the body temperature was ≥40.3 °C in these cats. For further details, see Spiri et al. [28]. In particular, the expression of proinflammatory cytokines such as IL-6 or TNF-α or anti-inflammatory cytokines as IL-10 could have been reduced in the treated cats, and the differences detected between the groups might have been more pronounced without anti-inflammatory treatment of the control cats.

After FCV Challenge II, the expression pattern of most cytokines resembled the pattern seen after FCV Challenge I, but fewer differences were seen between vaccinated and control cats, and the mRNA transcription levels were generally lower. Most probably, the control cats developed a strong adaptive immune response after FCV Challenge I, which prevented vast changes in cytokine mRNA transcription levels. The mRNA transcription levels of perforin and granzyme B followed a similar pattern as after FCV Challenge I. Interestingly, the mRNA transcription levels of IFN-β and IFN-ω were higher after FCV Challenge II compared to FCV Challenge I. As mentioned, some FCV strains seem to be able to evade the IFN-β and probably also the IFN-ω immune response by inhibiting the IFN promotor, which is characterised by low IFN mRNA transcription levels [63]. The higher mRNA transcription levels of IFN-β and IFN-ω after FCV Challenge II with FCV 27 could be indicative of an activation of the IFN-β and IFN- ω promotor. Therefore, unlike FCV 273, FCV 27 did not inhibit the IFN-β and IFN-ω promotor. This finding might indicate that FCV 273 was able to induce a host shut-off, where antiviral defence of the host is blocked by the virus and the host cells are forced to produce mainly viral proteins. The host shut-off immune evasion strategy has been documented for FCV [63,65,66], but whether the differences in virulence observed between the two FCV challenge isolates are based on this strategy remains inconclusive. The differences in mRNA expression levels of IFN-β and IFN-ω between the two FCV challenges were on a low level, and further in vitro and in vivo research is needed to address host shut-off mechanisms in FCV infections.

The present study measured the mRNA transcription levels of cytokines in blood. The clinical signs were mostly located in the upper respiratory tract and consisted mainly of oral ulcerations [28]. More pronounced cytokine changes could have been detected at the site of clinical manifestations. In tissue biopsies of cats suffering from feline chronic gingivostomatitis and FCV infection, increased mRNA transcription of Toll-like receptor 2, IL-1β, IL-6 and IFN-γ was detected [67]. In skin lesions of cats suffering from VS-FCV, upregulation of IL-10 and TNF-α was detected [68]. The collection of oral tissue samples is highly invasive and was thus not performed for the present study. However, the presence of FCV RNA in the peripheral blood in most of the cats in the present study [28] indicated that virus was present systemically; moreover, some of the cats had elevated body temperatures. Thus, changes in cytokine levels in the blood might have been expected, although possibly to a lesser extent than at the sites of primary infection and local manifestation of clinical signs.

Lymphocyte subsets consisting of T-helper cells (CD4^+^), cytotoxic T-cells (CD8^+^), B-cells (CD45R/B220^+^), and T-cells (CD5^+^) were assessed in vaccinated and control cats after FCV Vaccination I. In the first days after FCV Vaccination I, all lymphocyte subsets decreased transiently in the vaccinated cats and resulted in statistically significant lower CD8^+^ lymphocytes in vaccinated cats compared to control cats at day 4. It has been described that a haemotropic bacterial infection in cats can cause a transient recruitment and cell migration of lymphocytes from the blood to the injection site and the regional immune tissue [39]. CD8^+^ lymphocytes are cytotoxic T cells and are important for the defence against intracellular pathogens such as viruses. The decrease in CD8^+^ lymphocytes might therefore represent the cell migration and the recruitment of cytotoxic T-cells, leading to migration from blood to the regional lymph nodes. For CD4^+^, CD8^+^, and CD5^+^ lymphocytes, peak values were detected at days 27 and 40 in all vaccinated cats, which could represent the building of immunological memory after the second injection of FCV Vaccination I. Interestingly, peak numbers of B-cells (CD45R/B220^+^ lymphocytes) detected at day 13 after FCV Vaccination I coincide with the first detection of FCV antibodies in the vaccinated cats. Furthermore, a second B-cell peak was detected at day 34 (13 days after the second vaccine injection) and coincided with the highest FCV titres measured by IFA during the whole observation period after FCV Vaccination I. Some variation over time in the absolute number of lymphocyte subsets of vaccinated and control cats was detected. This variation reflected most probably the maturation of the immune system of the juvenile cats; the vaccination period started at 15 weeks of age. Juvenile cats have higher T- and B-cell absolute numbers than adult cats, and this might explain the lower numbers in all subsets detected at day 200 [69].

In the early phase after FCV Challenge I, all lymphocyte subsets (CD4^+^, CD8^+^, CD45R/B220^+^, CD5^+^, and CD5^+^/MHCII^+^) in all cats decreased in absolute numbers and returned to the preinfectional level by day 22 after challenge infection, except for CD4^+^/CD25^+^. The transient decrease in lymphocyte subsets reflected the decrease in absolute lymphocyte counts observed in all cats after FCV Challenge I [28]; this might represent again the cell migration from blood to the regional lymph nodes as discussed for FCV Vaccination I. However, the extent of the decrease was much greater after FCV Challenge I compared to FCV Vaccination I. At day 6 after the experimental FCV infection, the vaccinated cats had significantly higher CD5^+^ lymphocytes and CD8^+^ lymphocytes. CD5 is a marker for feline T-lymphocytes, and CD8^+^ cells represent cytotoxic T-cells. Cytotoxic T-cells seem to be important in vaccine-induced immunity against FCV. In a study in which PBMCs from FCV-vaccinated cats were restimulated in vitro, only inactivated FCV caused the proliferation of CD8^−^ and CD8^+^ lymphocytes, whereas infectious FCV caused a suppression of CD8^+^ lymphocyte numbers [64]. A decrease in CD8^+^ lymphocytes was observed in both groups immediately after FCV infection, but the decrease was less pronounced in the vaccinated than in the unvaccinated cats. CD4^+^/CD25^+^ lymphocytes did not follow the same pattern as the other lymphocyte subsets, and some cyclical variance was detected over time. The cyclical appearance of CD4^+^/CD25^+^ lymphocytes could not be correlated with either FCV RNA loads shed from the oropharynx, FCV RNA loads in blood, or with peak values of the acute phase protein serum-amyloid A, which were all described previously [28]. Additionally, no correlation could be detected with proinflammatory cytokines such as IL-6 or TNF-α and IL-10—a cytokine produced, among others, by T-regulatory cells [54]. CD4^+^/CD25^+^ lymphocytes represent activated T-helper cells, and a subpopulation represents T-regulatory cells that limit immune response and prevent excessive tissue damage. The anti-inflammatory treatment of some cats of the control group in the early phase of FCV Challenge I might have also influenced lymphocyte subsets, and the differences between the groups might have been more marked in the absence of treatment.

Of note, the total lymphocyte number was significantly lower in the control cats at day 6 [28]; this should be taken into account when interpreting the lymphocyte subsets, but the significant difference between the groups seen for CD8^+^ and CD5^+^ lymphocytes could not be explained solely by the lower lymphocyte number as other lymphocyte markers such as CD4^+^, CD45R/B220^+^, and CD5^+^/MHCII^+^ were not significantly different between the groups. After FCV Challenge II, the changes in the lymphocyte subsets and differences between the groups were similar, but to a lesser extent, compared to FCV Challenge I. This finding of more moderate changes after Challenge II compared to Challenge I correlates with the clinical presentation of the cats after FCV Challenge II, when no clinical signs and lower FCV RNA loads in the oropharynx were detected compared to Challenge I and no FCV RNA was detected in the blood. We assume that the FCV Challenge I also resulted in an immunisation and the development of a strong FCV-specific adaptive immune response in the control cats.

PBMCs of cats vaccinated with FCV F9 released IFN-γ upon stimulation with FCV F9 and FCV 273 in the ELISpot assay. This indicated that a cellular crossimmunity against FCV 273 was developed after FCV F9 vaccination. The ELISpot assay permits a quantitative analysis of a biological function on the single-cell level. IFN-γ is the hallmark cytokine of the Th1 pathway, which activates CD4^+^ T-helper cells upon antigen presentation on MHCII molecules, and IFN-γ is also produced by CD8^+^ effector T-cells, which are responsible for the killing of virus-infected cells [54]. Thus far, no study has described the use of the IFN-γ ELISpot assay in conjunction with FCV. A study from Tham and Studdert 1987 [25] investigated cell-mediated immunity following vaccination with inactivated FCV vaccines. A stimulation index of ConA-induced lymphocyte blastogenesis was calculated, and a high stimulation index in all vaccinated cats was detected after the first vaccination; in three out of four cats, an anamnestic lymphocyte blastogenesis was found after the second vaccination. In another study, FCV-specific CD4^+^ cells were found in the spleen of FCV-vaccinated cats [26]. These findings, together with the results of the present study, indicate that cellular immune mechanisms also play a role in building crossimmunity after FCV vaccination. Interestingly, similar crossimmunity was not detected in the humoral pathway as no crossneutralising antibodies were detected after FCV vaccination; although according to early studies, FCV F9 had been selected as a vaccine virus strain due to its broad neutralisation activity [13]. Our data suggest that different epitopes might be involved in stimulating either cellular or humoral immunity.

An anamnestic and high cell-mediated immune response was detected in the vaccinated cats during the early phase of the first experimental FCV infection. The MLV F9 vaccination was therefore beneficial in stimulating early and extensive cell-mediated immunity against a heterologous FCV infection. This finding is consistent with a previously published report where, after FCV challenge, the anamnestic lymphocyte blastogenesis occurred earlier in vaccinated than in control cats [25]. In the present study, hardly any IFN-γ-releasing PBMCs were detected in the control cats upon stimulation with FCV 273 before FCV Challenge I. After FCV Challenge I, however, the number of IFN-γ-releasing PBMCs increased significantly, indicating that FCV 273 infection induced a cellular immune response in the control cats. However, the combination of FCV F9 vaccination and FCV 273 infection induced a higher cellular immune response compared to FCV 273 infection alone as the median number of IFN-γ-releasing PBMCs was consistently higher in the vaccinated cats compared to the control cats over the whole observation period of FCV Challenge I. Before the second challenge, IFN-γ-releasing PBMCs were observed upon stimulation with FCV 27, and neutralising antibodies were detected in all cats, indicating both humoral and cellular crossimmunity. Even though the second challenge caused similar changes in lymphocyte subsets and cytokine mRNA transcription levels, the magnitude of the changes was less, and fewer differences between the groups were found. This is most probably due to the pre-existent crossimmunity. A similar finding was described previously where a previous infection with one FCV strain lessened clinical signs after subsequent exposure to a heterologous FCV strain [70]. Of note, after FCV Challenge II, no clinical signs and no FCV RNA in blood were detected in any of the cats [28]. It is likely therefore that the first experimental infection conferred crossimmunity against the second experimental infection.

Both challenge viruses, namely FCV 273 and FCV 27, were tested for susceptibility to virus neutralisation by sera raised against the FCV vaccine virus strain (FCV F9). Serum raised in SPF cats following infection of FCV F9 did not neutralise FCV 273, but FCV 27 was neutralised at a low titre. This finding could explain why the two experimental infections had different outcomes; however, FCV F9-vaccinated cats displayed less severe clinical signs, had a shorter duration of RNAemia and had lower viral loads shedding from the oropharynx after the challenge with FCV 273, as described elsewhere [28]. These findings indicate a beneficial effect of the MLV FCV F9 vaccine. In addition, the FCV infection with FCV 273 stimulated an extensive and broad immunity against a subsequent challenge with another FCV isolate.

## 5. Conclusions

Vaccination with a modified-live FCV F9 vaccine induced cellular, but not humoral, crossimmunity in SPF cats against one field FCV strain, indicating that different viral epitopes are involved. The magnitude of the humoral immune response induced by FCV F9 vaccination was highly variable between individuals, and, in the absence of maternal antibodies, high antibody titres were detected in some cats after a single FCV F9 vaccine injection. A Th1-directed immune response was elicited in cats vaccinated with the FCV F9 vaccine. The first experimental infection with a current FCV field strain induced humoral and cellular crossreactivity against the second challenge virus—a different current FCV field strain—and humoral crossimmunity against the vaccine strain FCV F9, as well as, to a lesser degree, an unrelated Swiss VS-FCV strain in most cats. Even though adaptive immunity was already present in the vaccinated cats, innate immune mechanisms still seem to play an important role in the early reaction against FCV infection. Cell-mediated FCV vaccine immunity and host-dependent factors influencing the magnitude of the humoral and cellular immune response should be further investigated, and the differences between inactivated and modified-live vaccines should be addressed in the future. The ability of some FCV strains to inhibit an innate antiviral immune response, by inhibition of the IFN-β promotor, might explain some differences in virulence between certain FCV isolates, and further research should be prompted to this direction.

## Figures and Tables

**Table 1:** Timepoints of blood collections for flow cytometry, cytokines, immunofluorescence assays (IFA), virus neutralisation, and peripheral blood mononuclear cells (PBMCs) collection for enzyme-linked immunospot (ELISpot) assay after FCV Vaccination I and II and FCV Challenges I and II (days).

	FCV Vaccination I *	FCV Challenge I	FCV Vaccination II	FCV Challenge II
Flow cytometry	−1, 4, 7, 13, 20, 22, 27, 34, 40, 48, 55, 62, 69, 76, 82, 90, 96, and 200 after injection I	−1, 1, 3, 6, 9, 13, 16, 22, 29, 36, 43, 50, 56, 63, 71, 78, 85, 92, 99, 106, 134, and 162	Not performed	−1, 1, 3, 6, 9, 13, 16, 22, 29, 36, and 44
Cytokines	−1, 4, 7, 13, 20, 22, 27, and 34 after injection I	−1, 1, 2, 3, 6, 9, 13, 16, and 22	Not performed	−1, 1, 2, 3, 6, 9, and 13
IFA	−24, 4, 7, 13, 27, 34, 40, 55, 69, 82, 96, 111, 133, 153, and 174 after injection I	−1, 1, 3, 6, 9, 13, 16, 22, 36, 50, 63, 78, 92, 106, 134, 162, 191, 204, 281, and 309	−1, 1, 2, 3, 8, and 34	−1, 3, 29, and 56
Virus neutralisation	−1, 13, 34, and 214 after injection I	−1, 6, 13, and 154	−1 and 34	−1 and 36
PBMC collection	−29, 22, and 92 after injection I	−5, 2, 8, 24, 38, 55, 113, and 204	Not performed	−4 and 38

* FCV Vaccination I consisted of two injections (injections I and II) 21 days apart.

**Table 2:** Endpoint neutralisation titres of cats of the vaccine and the control group, respectively. The titre is the reciprocal of the serum dilution where 50% or more of the wells show a CPE.

Day 154 after FCV Challenge I/Endpoint Titration				
	Cat ID	FCV F9	FCV 273	FCV 27
Vaccinated	JJG4	1215	1215	135
	JJG6	1215	1215	135
	JJH3	**3645**	**3645**	135
	JJI1	**10,935**	**32,805**	**3645**
	JJI2	**>98,415**	**3645**	1215
Control	JJF1	135	**10,935**	1215
	JJG3	15	1215	45
	JJH2	**3645**	**32,805**	405
	JJI3	405	**10,935**	1215
	JJI4	135	**10,935**	45

Endpoint titres >1215 are shown in bold.

**Table 3:** Endpoint neutralisation titres of cats of the vaccine and the control group, respectively. The titre is the reciprocal of the serum dilution where 50% or more of the wells show a CPE.

Day 36 after FCV Challenge II/Endpoint Titration		
	Cat ID	FCV 27
Vaccinated	JJG4	**3645**
	JJG6	**3645**
	JJH3	1215
	JJI1	**3645**
	JJI2	1215
Control	JJF1	**10,935**
	JJG3	**3645**
	JJH2	**3645**
	JJI3	**98,415**
	JJI4	1215

Titres >1215 are shown in bold.

## Data Availability

All available data are presented in this manuscript.

## Appendix A

**Table A1:** Results of Friedman and Dunn’s post test of cytokine mRNA transcription levels after FCV Vaccination I.

Cytokine	Change over Time (Friedman and Dunn’s Post Test) Vaccinated Group	Change over Time (Friedman and Dunn’s Post Test) Control Group
IL-10	P_F_ = 0.0255 Day 20/Day 34 ↑ (P_D_ = 0.0093)	P_F_ = 0.032 Day 20/Day 34 ↑ (P_D_ = 0.016)
MX1	P_F_ = 0.004 Day 4 ↑/Day 27 (P_D_ = 0.0443) Day 4 ↑/Day 34 (P_D_ = 0.0443)	Not significant
IFN-γ	P_F_ = 0.0155 Day 13 ↑/Day 22 (P_D_ = 0.0093)	Not significant
IL-12p40	P_F_ = 0.0077 Day 20 ↑/Day 34 (P_D_ = 0.0016)	P_F_ = 0.0037 Day 20 ↑/Day 34 (P_D_ = 0.0009)
IL-4	P_F_ = 0.0067 Day 7/Day 22 ↑ (P_D_ = 0.0016)	P_F_ = 0.0003 Day 4/Day 22 ↑ (P_D_ = 0.0093) Day 4/Day 27 ↑ (P_D_ = 0.0443) Day 4/Day 34 ↑ (P_D_ = 0.016) Day 7/Day 22 ↑ (P_D_ = 0.0269) Day 7/Day 34 ↑ (P_D_ = 0.0443)
IL-6	-	-
TNF-α	P_F_ = 0.0082	P_F_ = 0.0172 Day 13 ↑/Day 34 (P_D_ = 0.0269)
IFN-ω	-	-
Perforin	P_F_ = 0.044 Day 22/Day 27 ↑ (P_D_ = 0.016)	P_F_ = 0.029 Day 4 ↑/Day 34 (P_D_ = 0.016)
Granzyme B	P_F_ = 0.0272	-

**Table A2:** Results of the Friedman and Dunn’s post test of lymphocyte subsets after FCV Vaccination I.

Lymphocyte Subset	Change over Time (Friedman and Dunn’s Post Test) Vaccinated Group	Change over Time (Friedman and Dunn’s Post Test) Control Group
CD5	PF < 0.0001 Day 4↓/Day 27 (P_D_ = 0.023) Day 7↓/Day 27 (P_D_ = 0.0463) Day 13↓/Day 27 (P_D_ = 0.0367) Day 200↓/Day 27 (P_D_ = 0.0291)	P_F_ = 0.0368 Day 48/Day 90↑ (P_D_ = 0.0463)
CD45	P_F_ = 0.0001 Day −1↑/Day 22 (P_D_ = 0.023) Day 20↑/Day 22 (P_D_ = 0.0367) Day 34↑/Day 22 (P_D_ = 0.0463) Day 4↓/Day 34 (P_D_ = 0.0023) Day 200↓/Day 34 (P_D_ = 0.0111)	P_F_ = 0.0007
CD4	P_F_ = 0.0001 Day 40↑/Day 200 (P_D_ = 0.0142) Day 90↑/Day 200 (P_D_ = 0.0463)	P_F_ = 0.0038 Day 48/Day 90↑ (P_D_ = 0.0367)
CD8	P_F_ < 0.0001 Day 4↓/Day 40 (P_D_ = 0.0111) Day 7↓/Day 40 (P_D_ = 0.0181) Day 13↓/Day 40 (P_D_ = 0.0463) Day 22↓/Day 40 (P_D_ = 0.0142) Day 200↓/Day 40 (P_D_ = 0.023)	Not significant
CD4/CD8 ratio	P_F_ = 0.0003 Day 4↑/Day 40 (P_D_ = 0.0367) Day 7↑/Day 40 (P_D_ = 0.0086) Day 13↑/Day 40 (P_D_ = 0.0291) Day 22↑/Day 40 (P_D_ = 0.023)	Not significant

**Table A3:** Results of the Friedman and Dunn’s post test of IFN-γ-releasing PBMCs in vaccinated and control cats after FCV Vaccination I measured by ELISpot.

IFN-γ-Releasing PBMCs (ELISpot)	Change over Time (Friedman and Dunn’s Post Test) Vaccinated Group	Change over Time (Friedman and Dunn’s Post Test) Control Group
	P_F_ = 0.0293 P_D_ > 0.05 significance lost in the post test	P_F_ > 0.05

**Table A4:** Results of Friedman and Dunn’s post test of cytokine mRNA transcription levels after FCV Challenge I.

Cytokine	Change over Time (Friedman and Dunn’s Post Test) Vaccinated Group	Change over Time (Friedman and Dunn’s Post Test) Control Group
IFN-γ	P_F_ = 0.036 Day 1 ↑/Day 13 (P_D_ = 0.0221)	P_F_ = 0.0022 Day 1↑/Day 6 (P_D_ = 0.0221) Day1↑/Day 16 (P_D_ = 0.0137)
IL-12p40	P_F_ = 0.0222 Day 1 ↓/Day 22 (P_D_ = 0.0221)	P_F_ = 0.0123 Day 1 ↓/Day 2 (P_D_ = 0.0221)
IL-4	P_F_ = 0.0067 Day 1↑/Day 13 (P_D_ = 0.0084)	P_F_ = 0.0003 Day 1↑/Day 6 (P_D_ = 0.0006) Day 1↑/Day 9 (P_D_ = 0.035) Day 2↑/Day 6 (P_D_ = 0.0137)
IL-10	P_F_ = 0.027	P_F_ = 0.0335
TNF-α	P_F_ = 0.0036 Day 1↓/Day 3 (P_D_ = 0.0006) Day 1↓/Day 9 (P_D_ = 0.035)	P_F_ = 0.0126
IFN-α	P_F_ = 0.0387 Day 1↑/Day 3 (P_D_ = 0.035)	P_F_ = 0.0162 Day 1↑/Day 9 (P_D_ = 0.035) Day 1↑/Day 16 (P_D_ = 0.0084)
IFN-β	P_F_ = 0.0003 Day 1↑/Day 13 (P_D_ = 0.0137) Day 2↑/Day 3 (P_D_ = 0.035) Day 2↑/Day 9 (P_D_ = 0.035) Day 2↑/Day 13 (P_D_ = 0.0084)	P_F_ = 0.0063 Day 1/Day 9 (P_D_ = 0.0221)
MX1	P_F_ < 0.0001 Day 1↑/Day 13 (P_D_ = 0.0084) Day 1↑/Day 16 (P_D_ = 0.0137) Day 1↑/Day 22 (P_D_ = 0.0006) Day 3↑/Day 22 (P_D_ = 0.0084)	P_F_ < 0.0001 Day 1↑/Day 13 (P_D_ = 0.0137) Day 1↑/Day 16 (P_D_ = 0.0084) Day 1↑/Day 22 (P_D_ = 0.001) Day 3↑/Dy 16 (P_D_ = 0.035) Day 3↑/Day 22 (P_D_ = 0.0051)
Perforin	P_F_ < 0.0001 Day 1↓/Day 9 (P_D_ = 0.0018) Day 1↓/Day 13 (P_D_ = 0.0051) Day 2↓/Day 9 (P_D_ = 0.0221)	P_F_ = 0.0002 Day 1↓/Day 13 (P_D_ = 0.0221) Day 1↓/Day 16 (P_D_ = 0.0221) Day 1↓/Day 22 (P_D_ = 0.0221)
Granzyme B	P_F_ = 0.0005 Day 1↓/Day 13 (P_D_ = 0.035) Day 2↓/Day 13 (P_D_ = 0.0051) Day 2↓/Day16 (P_D_ = 0.035)	P_F_ = 0.0005 Day 1↓/Day 13 (P_D_ = 0.0221) Day 6↓/Day 13 (P_D_ = 0.035)

**Table A5:** Results of the Friedman and Dunn’s post test of lymphocyte subsets after FCV Challenge I.

Lymphocyte Subset	Change over Time (Friedman and Dunn’s Post Test) Vaccinated Group	Change over Time (Friedman and Dunn’s Post Test) Control Group
CD5	P_F_ < 0.0001 Day −1↑/Day 1 (P_D_ = 0.0017) Day −1↑/Day 3 (P_D_ = 0.008) Day −1↑/Day 6 (P_D_ = 0.0336) Day −1↑/Day 162 (P_D_ = 0.0409) Day 1↓/Day 78 (P_D_ = 0.0034) Day 3↓/Day 78 (P_D_ = 0.0151)	P_F_ < 0.0001 Day −1↑/Day 1 (P_D_ = 0.0496) Day 1↓/Day 22 (P_D_ = 0.0004) Day 3↓/Day 22 (P_D_ = 0.0027) Day 6↓/Day 22 (P_D_ = 0.0014) Day 1↓/Day 78 (P_D_ = 0.0409)
CD5/MHCII	P_F_ < 0.0001 Day −1↑/Day 1 (P_D_ = 0.0017) Day −1↑/Day 3 (P_D_ = 0.0151) Day −1↑/Day 6 (P_D_ = 0.0409) Day −1↑/Day 162 (P_D_ = 0.0276) Day 78↑/Day 1 (P_D_ = 0.0226) Day 1↓/Day 50 (P_D_ = 0.0496) Day 1↓/Day 56 (P_D_ = 0.0336) Day 1↓/Day 106 (P_D_ = 0.0099)	P_F_ < 0.0001 Day −1↑/Day 1 (P_D_ = 0.0496) Day 1↓/Day 22 (P_D_ = 0.0007) Day 3↓/Day 22 (P_D_ = 0.0042) Day 6↓/Day 22 (P_D_ = 0.0022 Day 1↓/Day 63 (P_D_ = 0.0496)
CD45	P_F_ < 0.0001 Day −1↑/Day 1 (P_D_ = 0.0226) Day −1↑/Day 3 (P_D_ = 0.0151) Day 1↓/Day 29 (P_D_ = 0.008) Day 1↓/Day 43 (P_D_ = 0.0122) Day 1↓/Day 50 (P_D_ = 0.0099 Day 1↓/Day 63 (P_D_ = 0.0409 Day 1↓/Day 78 (P_D_ = 0.0226 Day 3↓/Day 29 (P_D_ = 0.0052 Day 3↓/Day 43 (P_D_ = 0.008) Day 3↓/Day 50 (P_D_ = 0.0065) Day 3↓/Day 63 (P_D_ = 0.0276) Day 3↓/Day 78 (P_D_ = 0.0151) Day 6↓/Day 29 (P_D_ = 0.0336) Day 6↓/Day 43 (P_D_ = 0.0496) Day 6↓/Day 50 (P_D_ = 0.0409)	P_F_ < 0.0001 Day 1↓/Day 50 (P_D_ = 0.0122) Day 1↓/Day 56 (P_D_ = 0.008) Day 1↓/Day 63 (P_D_ = 0.0151) Day 3↓/Day 50 (P_D_ = 0.0276) Day 3↓/Day 56 (P_D_ = 0.0185) Day 3↓/Day 63 (P_D_ = 0.0336) Day 6↓/Day 50 (P_D_ = 0.0099) Day 6↓/Day 56 (P_D_ = 0.0065) Day 6↓/Day 63 (P_D_ = 0.0122)
CD4	P_F_ < 0.0001 Day −1↑/Day 1 (P_D_ = 0.0011) Day −1↑/Day 3 (P_D_ = 0.0052) Day −1↑/Day 6 (P_D_ = 0.0276) Day −1↑/Day 162 (P_D_ = 0.0226) Day 1↓/Day 78 (P_D_ = 0.008) Day 1↓/Day 106 (P_D_ = 0.0226) Day 3↓/Day 78 (P_D_ = 0.0336)	P_F_ < 0.0001 Day −1↑/Day 1 (P_D_ = 0.0226) Day 1↓/Day 22 (P_D_ = 0.0007) Day 3↓/Day 22 (P_D_ = 0.0042) Day 6↓/Day 22 (P_D_ = 0.0022) Day 1↓/Day 78 (P_D_ = 0.0226)
CD4/CD25	P_F_ < 0.0001 Day 6↓/Day 13 (P_D_ = 0.0099) Day 6↓/Day 16 (P_D_ = 0.0122) Day 6↓/Day 29 (P_D_ = 0.0034) Day 6↓/Day 106 (P_D_ = 0.0496) Day 6↓/Day 162 (P_D_ = 0.0276) Day 13↑/Day 63 (P_D_ = 0.0496) Day 29↑/Day 63 (P_D_ = 0.0185) Day 29↑/Day 71 (P_D_ = 0.0409)	P_F_ < 0.0001 Day 1↓/Day 13 (P_D_ = 0.0042) Day 1↓/Day 16 (P_D_ = 0.0014) Day 1↓/Day 29 (P_D_ = 0.0042) Day 1↓/Day 162 (P_D_ = 0.0052) Day 6↓/Day 13 (P_D_ = 0.0017) Day 6↓/Day 16 (P_D_ = 0.0005) Day 6↓/Day 29 (P_D_ = 0.0017) Day 6↓/Day 85 (P_D_ = 0.0496) Day 6↓/Day 162 (P_D_ = 0.0022) Day 16↑/Day 71 (P_D_ = 0.0226)
CD8	P_F_ < 0.0001 Day −1↑/Day 1 (P_D_ = 0.0027) Day −1↑/Day 3 (P_D_ = 0.008) Day −1↑/Day 6 (P_D_ = 0.0496) Day 50↑/Day 1 (P_D_ = 0.0276) Day 78↑/Day 1(P_D_ = 0.0042) Day 78↑/Day 3 (P_D_ = 0.0122)	P_F_ < 0.0001 Day 22↑/Day 1 (P_D_ = 0.0002) Day 22↑/Day 3 (P_D_ = 0.0009) Day 22↑/Day 6 (P_D_ = 0.0005) Day 22↑/Day 9 (P_D_ = 0.0151) Day 22↑/Day 162 (P_D_ = 0.0151)
CD4/CD8 ratio	P_F_ = 0.0003 Day 3↑/Day 29 (P_D_ = 0.0042) Day 3↑/Day 43 (P_D_ = 0.008) Day 3↑/Day 50 (P_D_ = 0.0336)	P_F_ < 0.0001 Day3↑/Day 22 (P_D_ = 0.0226) Day 3↑/Day 29 (P_D_ = 0.0122) Day 3↑/Day 36 (P_D_ = 0.0409) Day 3↑/Day 43 (P_D_ = 0.0276) Day 6↑/Day 22 (P_D_ = 0.0185) Day 6↑/Day 29 (P_D_ = 0.0099) Day 6↑/Day 36 (P_D_ = 0.0336) Day 6↑/Day 43 (P_D_ = 0.0226) Day 6↑/Day 134 (P_D_ = 0.0151)

**Table A6:** Results of the Friedman and Dunn’s post test of IFN-γ-releasing PBMCs in vaccinated and control after FCV Challenge I measured by ELISpot.

IFN-γ-Releasing PBMCs (ELISpot)	Change over Time (Friedman and Dunn’s Post Test) Vaccinated Group	Change over Time (Friedman and Dunn’s Post Test) Control Group
	No Friedman and Dunn’s post test possible (missing value)	P_F_ = 0.0001 Day −5↓/Day 55 (P_D_ = 0.0051) Day −5↓/Day 204 (P_D_ = 0.035) Day 2↓/Day 55 (P_D_ = 0.0018) Day 2↓/Day 204 (P_D_ = 0.0137)

**Table A7:** Results of Friedman and Dunn’s post test of cytokine mRNA transcription levels after FCV Challenge II.

Cytokine	Change over Time (Friedman and Dunn’s Post Test) Vaccinated Group	Change over Time (Friedman and Dunn’s Post Test) Control Group
IL-12p40	P_F_ = 0.0037 Day 1↓/Day2 (P_D_ = 0.0007)	P_F_ = 0.0234 Day 2↑/Day 9 (P_D_ = 0.0352)
IL-4	P_F_ = 0.0134 Day 1↑/Day 13 (P_D_ = 0.0108)	P_F_ = 0.0076 Day 1↑/Day 6 (P_D_ = 0.0108) Day 2↑/Day 6 (P_D_ = 0.0198)
IL-6	P_F_ = 0.008 Day 1↓/Day 13 (P_D_ = 0.0352) Day 2↓/Day 13 (P_D_ = 0.0058)	P_F_ = 0.0213 Day 2↓/Day 13 (P_D_ = 0.0058)
IL-10	P_F_ = 0.0421 Day 9↓/Day 13 (P_D_ = 0.0198)	Not significant
TNF-α	P_F_ = 0.0088 Day 1↓/Day 13 (P_D_ = 0.0198) Day 3↓/Day 13 (P_D_ = 0.0198)	Not significant
IFN-β	Not significant	Not significant
MX1	P_F_ = 0.0009 Day 1↑/Day 9 (P_D_ = 0.0198) Day 1↑/Day 13 (P_D_ = 0.0352) Day 2↑/Day 9 (P_D_ = 0.0352)	P_F_ = 0.0005 Day 1↑/Day 6 (P_D_ = 0.0108) Day 1↑/Day 13 (P_D_ = 0.0108) Day 2↑/Day 6 (P_D_ = 0.0198) Day 2↑/Day 13 (P_D_ = 0.0198)
Perforin	P_F_ = 0.0005 Day 1↓/Day 6 (P_D_ = 0.0198) Day 1↓/Day 13 (P_D_ = 0.0030) Day 2↓/Day 13 (P_D_ = 0.0352)	P_F_ = 0.0009 Day 2↓/Day 13(P_D_ = 0.0198) Day 3↓/Day 13 (P_D_ = 0.0030)
Granzyme B	P_F_ = 0.0106 Day 2↓/Day 13 (P_D_ = 0.0108)	P_F_ = 0.0024 Day 3↓/Day 6 (P_D_ = 0.0108) Day 3↓/Day 13 (P_D_ = 0.0352)

**Table A8:** Results of the Friedman and Dunn’s post test of lymphocyte subsets after FCV Challenge II.

Lymphocyte Subset	Change over Time (Friedman and Dunn’s Post Test) Vaccinated Group	Change over Time (Friedman and Dunn’s Post Test) Control Group
CD5	P_F_ = 0.0002 Day 1↓/Day 29 (P_D_ = 0.0075) Day 3↓/Day 29 (P_D_ = 0.011)	P_F_ = 0.0006 Day 1↓/Day 29 (P_D_ = 0.0329) Day 3↓/Day 29 (P_D_ = 0.011) Day 3↓/Day 36 (P_D_ = 0.0466)
CD5/MHCII	P_F_ = 0.0007 Day 1↓/Day 13 (P_D_ = 0.0329) Day 1↓/Day 29 (P_D_ = 0.0466)	P_F_ = 0.0046 Significance was lost in the post test
CD45	P_F_ = 0.0001 Day −1↑/Day 3 (P_D_ = 0.0329) Day 3↓/Day 36 (P_D_ = 0.023) Day 3↓/Day 44 (P_D_ = 0.011)	P_F_ < 0.0001 Day −1↑/Day 3 (P_D_ = 0.0329) Day 1↓/Day 36 (P_D_ = 0.0329) Day 1↓/Day 44 (P_D_ = 0.0034) Day 3↓/Day 36 (P_D_ = 0.0034) Day 3↓/Day 44 (P_D_ = 0.0003) Day 6↓/Day 44 (P_D_ = 0.0466) Day 13↓/Day 44 (P_D_ = 0.0466)
CD4	P_F_ = 0.0002 Day −1↑/Day 1 (P_D_ = 0.0466) Day −1↑/Day 3 (P_D_ = 0.016) Day 1↓/Day 29 (P_D_ = 0.023) Day 3↓/Day 13 (P_D_ = 0.0329) Day 3↓/Day 29 (P_D_ = 0.0075)	P_F_ = 0.0002 Day 1↓/Day 29 (P_D_ = 0.0329) Day 3↓/Day 29 (P_D_ = 0.0075) Day 3↓/Day 44 (P_D_ = 0.023)
CD4/CD25	P_F_ = 0.0004 Day 1↓/Day 22 (P_D_ = 0.0466) Day 22↑/Day 44 (P_D_ = 0.0034)	P_F_ < 0.0001 Day −1↓/Day 16 (P_D_ = 0.0051) Day −1↓/Day 22 (P_D_ = 0.0075) Day 16↑/Day 29 (P_D_ = 0.0329) Day 22↑/Day 29 (P_D_ = 0.0466)
CD8	P_F_ = 0.0029 Day −1↑/Day 1 (P_D_ = 0.023)	P_F_ < 0.0001 Day 1↓/Day 36 (P_D_ = 0.0466) Day 1↓/Day 44 (P_D_ = 0.016) Day 3↓/Day 36 (P_D_ = 0.0329) Day 3↓/Day 44 (P_D_ = 0.011)
CD4/CD8 ratio	P_F_ = 0.0314 Day 1↑/Day 22 (P_D_ = 0.0034)	P_F_ = 0.0027 Day 1↑/Day 22 (P_D_ = 0.0466) Day 3↑/Day 22 (P_D_ = 0.0023)

**Table A9:** Results of the Friedman and Dunn’s post test of IFN-γ-releasing PBMCs in vaccinated and control after stimulation with FCV 27 after FCV Challenge II.

IFN-γ-Releasing PBMCs (ELISpot)	Change over Time (Mann–Whitney U) Vaccinated Group	Change over Time (Mann–Whitney U) Control Group
	Not significant	Not significant
